# Dendritic polarizing agents for DNP SENS†
†Electronic supplementary information (ESI) available. See DOI: 10.1039/c6sc03139k



**DOI:** 10.1039/c6sc03139k

**Published:** 2016-08-22

**Authors:** Wei-Chih Liao, Ta-Chung Ong, David Gajan, Florian Bernada, Claire Sauvée, Maxim Yulikov, Margherita Pucino, Roman Schowner, Martin Schwarzwälder, Michael R. Buchmeiser, Gunnar Jeschke, Paul Tordo, Olivier Ouari, Anne Lesage, Lyndon Emsley, Christophe Copéret

**Affiliations:** a Department of Chemistry and Applied Biosciences , ETH Zürich , Vladimir-Prelog-Weg 1-5 , 8093 Zürich , Switzerland . Email: ccoperet@inorg.chem.ethz.ch; b Centre de RMN à Très Hauts Champs , Institut de Sciences Analytiques (CNRS/ENS Lyon/UCB Lyon 1) , Université de Lyon , 69100 Villeurbanne , France; c Aix-Marseille Univ , CNRS , ICR UMR 7273 , Marseille , 13013 , France; d Institut für Polymerchemie , Universität Stuttgart , Pfaffenwaldring 55 , D-70569 Stuttgart , Germany; e Institut des Sciences et Ingénierie Chimiques , Ecole Polytechnique Fédérale de Lausanne (EPFL) , 1015 Lausanne , Switzerland

## Abstract

Dynamic Nuclear Polarization Surface Enhanced NMR Spectroscopy (DNP SENS) is an effective method to significantly improve solid-state NMR investigation of solid surfaces. The presence of unpaired electrons (polarizing agents) is crucial for DNP, but it has drawbacks such as leading to faster nuclear spin relaxation, or even reaction with the substrate under investigation. The latter can be a particular problem for heterogeneous catalysts. Here, we present a series of carbosilane-based dendritic polarizing agents, in which the bulky dendrimer can reduce the interaction between the solid surface and the free radical. We thereby preserve long nuclear *T*′_2_ of the surface species, and even successfully enhance a reactive heterogeneous metathesis catalyst.

## Abstract 2

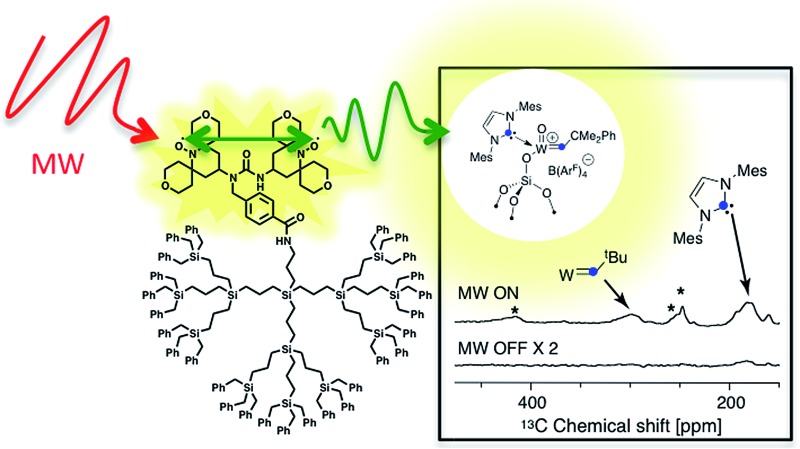
Dendrimer-shielded polarizing agents for the application of DNP SENS to reactive surfaces.

## Introduction

Solid-state nuclear magnetic resonance (NMR) spectroscopy is one of the most powerful tools used to probe surface structures at the atomic level.^1^ However, the application of NMR is impeded by its inherently low sensitivity, which is compounded by the small fraction of sites of interest present on solid surfaces. Dynamic Nuclear Polarization Surface Enhanced NMR Spectroscopy (DNP SENS) has recently emerged as an effective method greatly increasing sensitivity for surface species.^2–7^ It relies on a transfer of the large electron polarization of a paramagnetic dopant to the nearby nuclei.^8^ A persistent radical, *e.g.* a nitroxide biradical, is typically used as the polarizing agent (PA). The PA is usually dissolved in a glass-forming solvent and then introduced onto the solid under analysis by incipient wetness impregnation.^2^ The sample is then usually cooled to 100 K or lower, and microwaves are applied to saturate the electron paramagnetic resonance (EPR) transition, which induces, predominantly through the cross effect mechanism,^9–11^ the transfer of the electron polarization to the protons of the surrounding solvent molecules. This is typically followed by a cross-polarization^12^ (CP) step to the heteronuclei on the solid surface, resulting in a significant sensitivity increase of their NMR resonances. With sensitivity gains of one to two orders of magnitude, the signal averaging time needed is dramatically reduced, and the detailed characterization of surface species becomes possible. Notably, the improvement in sensitivity enables advanced two-dimensional (2D) NMR experiments that are not practicable using conventional solid-state NMR spectroscopy.^6,13–20^

Over the last decade, much effort has been devoted to developing free radicals that yield larger DNP enhancements. In 2004, Hu *et al.* ^21^ demonstrated that binitroxide radicals were highly efficient for cross effect DNP due to large intramolecular dipolar couplings between the two tethered nitroxide radicals. In 2012, Zagdoun *et al.* ^22^ showed that binitroxide radicals could be engineered to have longer *T*_1e_, based on bulky, rigid skeletons,^23^ and that this led to unprecedented DNP enhancements. Michaelis *et al.*,^24^ Kubicki *et al.* ^25^ and Sauvée *et al.* ^26^ have recently reported comprehensive studies, in which many binitroxide radicals were investigated in order to rationalize the effect of biradical structures on DNP performances. Note that hydrophobic radicals have been solubilized successfully in aqueous solutions by incorporating them into cyclic oligosaccharides or micelles.^27,28^ Using the most recently developed binitroxides, including TEKPol- and AMUPol-like biradicals, DNP enhancements of between 200 and 300 have been achieved in bulk solutions at 9.4 T and 100 K. These PAs have been successfully employed to polarize solutions to characterize the surfaces of a wide range of materials including single-site catalysts,^16^ silica-based materials,^29–31^ zeolites,^14,32,33^ metal oxides,^19,20^ supported nanoparticles^34^ and colloidal nanoparticles.^35^

In this approach, however, free radicals are inevitably in proximity to the surface, which potentially leads to paramagnetic quenching of the NMR signal, to faster transverse relaxation of the surface nuclei, or most problematically to reaction with the substrate.^36,37^ Most DNP studies reported so far have focused on relatively stable materials, with only a few targeting the characterization of reactive surface species.^16,29,34,38^ In particular, in the field of heterogeneous catalysis, Lewis acidic metal centers can interact with the free radicals.^39–43^ For instance, bulky PAs are needed to probe the structure of active sites in zeolites because they cannot enter the porous network and interact with the active sites.^33^ It would thus be desirable to develop polarizing agents that are compatible with non-porous and large-pore-size reactive materials.

**Scheme 1:**
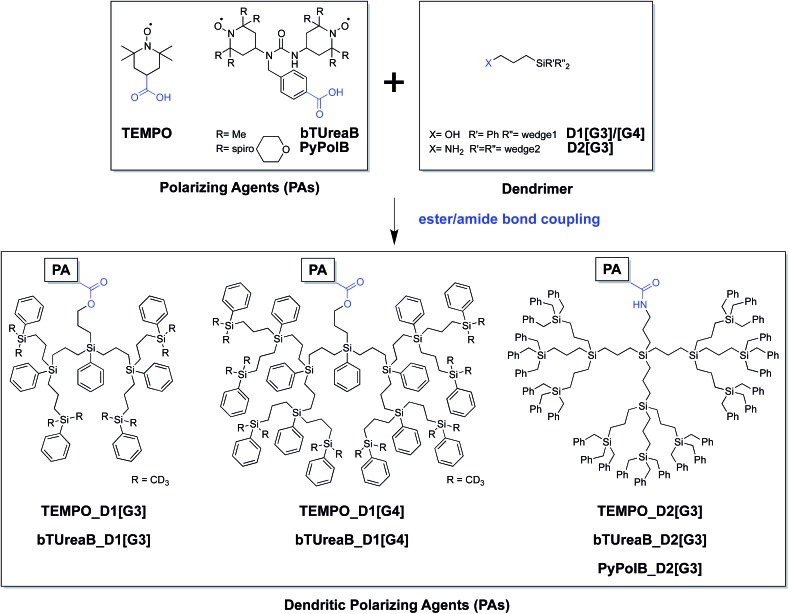
Convergent synthetic route of the dendritic PAs.

Here we present the syntheses (Scheme 1) and EPR characterization of dendritic PAs combining a monoradical, 2,2,6,6-tetramethylpiperidine-1-oxyl (TEMPO), or biradicals (bTUrea and PyPol)^44^ with core-functionalized hydrophobic carbosilane dendrimers.^45,46^ We show in particular that the dendritic PA with the bulkiest and most rigid structure displays the minimum interference with the surface sites, and preserves the coherence life time (*T*′_2_) and has the best DNP SENS performance (*ε*_^13^C CP_ = 128). We also illustrate the advantage of dendritic PAs by characterizing a reactive material, a recently developed cationic heterogeneous alkene metathesis catalyst.^47^ Notably, for this particular catalyst, the currently favored “conventional” biradical, TEKPol, showed no DNP enhancement.

## Results and discussion

### General description of syntheses

**Scheme 2:**
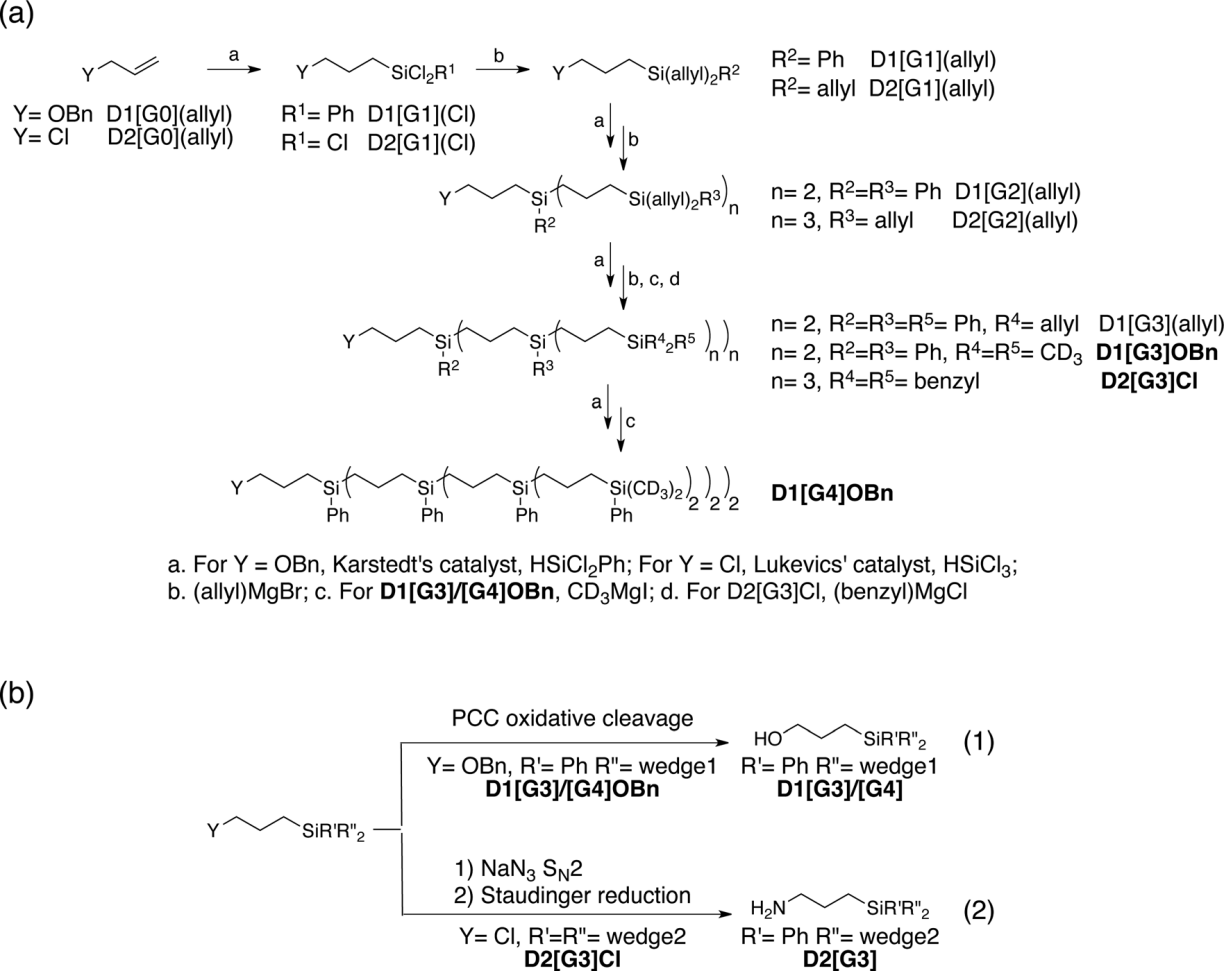
(a) Divergent synthetic route of the dendrimers. (b) Syntheses of the anchor groups on the dendrimers.

A convergent synthetic route for the dendritic PAs was adopted, in which the monoradical or biradical is introduced onto the dangling OH or NH_2_ functionalities at the core of the dendrimer (Scheme 1). For the radical, commercially available 4-carboxy TEMPO was used, and the biradicals **bTUreaB** and **PyPolB** were prepared by adapting the previously reported procedure.^44^ For the dendrimer, carbosilane dendritic backbones were chosen due to their apolar and inert nature. A divergent repetitive Pt(0)-catalyzed hydrosilylation and Grignard displacement strategy^45,46^ was utilized to construct the dendrimers (Scheme 2).

Two dendrimer designs were explored: **D1** with two dendritic wedges and **D2** with three dendritic wedges (Scheme 1 and 2a). From allylbenzyl ether, **D1** was built by repetitive hydrosilylation with (dichloro)phenylsilane catalyzed by Karstedt's catalyst^48^ followed by reaction with allylmagnesium bromide. Trideuteriomethyl-magnesium iodide (CD_3_MgI) was used to install the end groups to prevent fast electron and nuclear relaxations promoted by the rotations of methyl groups (–CH_3_) at 100 K.^25,49,50^ The benzyl group was then removed by homogeneous oxidative cleavage to generate the OH anchor group (Scheme 2b). We note that the typical Pd/C catalyzed reductive cleavage failed, suggesting the inaccessibility of the benzyl ether on the dendrimer to the Pd/C surface. **D1** with generation 3 and generation 4 (**D1[G3]/[G4]**) were synthesized, and their respective hydrodynamic diameters are around 1.7 nm and 2.3 nm as measured by DOSY NMR (ESI†).

For **D2**, the same divergent synthetic strategy was utilized. Trichlorosilane was used instead of dichlorophenylsilane to afford three branches at the diverging points. Since allylbenzyl ether was reduced by silyl hydride at the oxygen site, 3-chloro-1-propene was thus used instead, and Lukevic's catalyst^51^ was used to catalyze the hydrosilylation. The alkyl chloride at the core of **D2** was found to exchange with CD_3_MgI and result in an insoluble gel product. Benzylmagnesium chloride was thus used instead to form the end groups of **D2**. Further azide substitution and Staudinger reduction afforded the NH_2_ anchor group (Scheme 2b). Notably, the substitution step took days to complete, indicating that the amine site is probably buried inside the dendrimer. **D2** with generation 3 (**D2[G3]**) was synthesized, and its hydrodynamic diameter is around 2.3 nm (ESI†), which is larger than **D1[G3]**.

Finally, dendritic PAs were readily formed *via* conventional ester/amide bond couplings. However, we noted that **PyPolB** could not be connected to OH-anchored dendrimers (**D1[G3]/[G4]**) possibly due to the large steric hindrance of the dendrimer and lower reactivity of OH group.

### EPR properties

Obtaining good DNP enhancements requires a homogeneous distribution of PAs in the frozen glassy matrix. Aggregation or crystallization can generate an inhomogeneous distribution of free radicals leading to a lower DNP efficiency.^8,52^ As macromolecules are known to self-assemble/aggregate,^53^ the EPR line widths and relaxation time constants (*T*_1e_ and *T*_2e_) of the dendritic PAs were measured. First, the dendritic monoradicals (**TEMPO-D1[G3]/D1[G4]/D2[G3]**) and the non-dendritic analogue (**TEMPO-C3**, Fig. S1†) were separately prepared as 30 mM 1,1,2,2-tetrachloroethane (TCE) solutions. Their EPR profiles (Fig. S2†) were measured on a Bruker X band continuous wave (CW) EPR spectrometer at 100 K. The average inter-radical distances were estimated by measuring the EPR line widths, which were found to be the same for all samples (11.7 Gauss), suggesting an average interradical distance of at least 1.8 nm in the frozen matrix.^54^ Secondly, we investigated the electron relaxation time constants (*T*_1e_ and *T*_2e_) as an alternative probe of the distribution of biradicals since their EPR profiles were significantly perturbed by strong intramolecular electron–electron dipolar couplings. The dendritic biradicals (**bTUreaB-D1[G3]/D1[G4]/D2[G3]** and **PyPolB-D2[G3]**) and the non-dendritic analogue (**bTUreaB-C3** and **PyPolB-C3**, Fig. S1†) were separately prepared as 16 mM TCE solutions. *T*_1e_ and *T*_2e_ were measured on a Bruker W band pulsed EPR spectrometer at 100 K. All biradicals had similar *T*_1e_ and *T*_2e_ (Table 1). Both the measured line widths and relaxation time constants indicate similar average inter-monoradical and -biradical distances in dendritic and non-dendritic PAs, suggesting the absence of significant aggregation induced by the dendrimers.

**Table 1:** *T*_1e_ and *T*_2e_ of the dendritic and non-dendritic PAs^*a*^

	*T*_1e_ (ms)	*T*_2e_ (ns)
**bTUreaB-C3**	26	411
**bTUreaB-D1[G3]**	25	431
**bTUreaB-D1[G4]**	25	410
**bTUreaB-D2[G3]**	28	424
**PyPolB-C3**	82	1518
**PyPolB-D2[G3]**	82	1607

^*a*^Errors were estimated to be approximately 5% as spectra with good signal-to-noise ratio were recorded for all samples. See ESI for details.

### Magic-angle-spinning (MAS) DNP performance

The MAS DNP performance of the dendritic monoradical **TEMPO-D1[G3]** was assessed as a 30 mM solution in TCE. The mixture was frozen at 100 K, and the solvent enhancement (*ε*_^13^C CP_)^55^ was found to be 6, with a proton build-up time (*T*_DNP_) of 2.5 s on a Bruker 400 MHz (9.4 T) DNP spectrometer (Table 2). A possible explanation to this low enhancement is that since the cross effect is the most efficient DNP mechanism at 100 K, and requires strong dipolar coupling between two unpaired electrons, the steric hindrance of the dendrimer likely decreases the probability of free radical pairs to be at an optimal distance (∼1.3 nm)^21^ to fulfill the matching condition of the cross effect. However, this difference in free radical distance distribution is seemingly hard to be detected from their EPR line widths (11.7 Gauss on the X band CW EPR spectrometer). The dendritic biradicals were separately prepared as 16 mM solutions in TCE and frozen at 100 K. On Bruker 400 MHz and 600 MHz DNP spectrometers, the enhancement values for the dendritic PAs with **bTUreaB** were around 45 and 10, respectively; and for **PyPolB-D2[G3]**, the values were 128 and 29, respectively (Table 2). *T*_DNP_ for **bTUreaB** PAs were about 1.5 s on a 400 MHz spectrometer, and around 2.2 s and 2.5 s for **PyPolB-D2[G3]** on 400 MHz and 600 MHz DNP spectrometers, respectively (Table 2). The enhancements of dendritic biradicals were essentially the same as of the non-dendritic biradicals in TCE.^25^ We conclude that the dendrimer does not adversely affect the biradical conformations or glass forming properties of the solvent. In particular the dendritic PA, **PyPolB-D2[G3]** is found here to perform at least as well as AMUPol in TCE.^25^ Additionally, we noted the presence of ^13^C NMR signals of the dendrimer in bulk solutions (Fig. S7†). This is presumably due to the large size of the dendrimer, some part of the dendritic structure experiencing less or no paramagnetic bleaching by the unpaired electrons.

**Table 2:** MAS DNP properties of dendritic PAs in bulk solutions^*a*^

	*ε*_^13^C CP_ ^*a*^ (400/600 MHz)	*T*_DNP_ (s) (400/600 MHz)
**TEMPO-D1[G3]**	6/—	2.5(1)/—
**bTUreaB-D1[G3]**	41/10	1.5(1)/—
**bTUreaB-D1[G4]**	45/10	1.5(1)/—
**bTUreaB-D2[G3]**	48/13	1.5(1)/4.6(1)^*b*^
**PyPolB-D2[G3]**	128/29	2.2(1)/2.5(1)

^*a*^The error is within 8% calculated based on the signal-to-noise ratio.

^*b*^Presumably due to poor glass formation.

### Using dendritic PAs in DNP SENS on representative solid materials

**Fig. 1:**
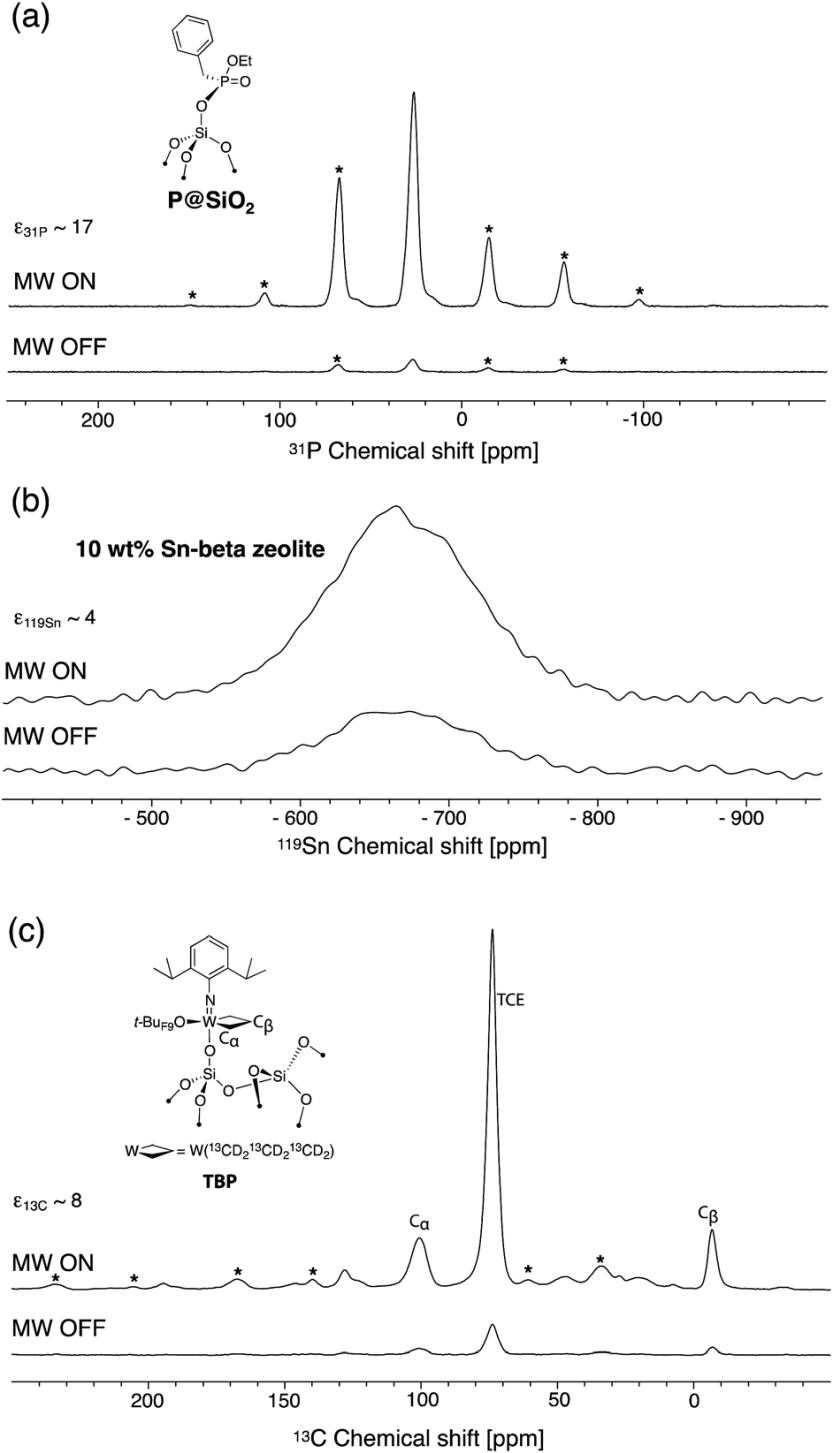
DNP (a) ^31^P CP Hahn-echo spectra of **P@SiO_2_**, (b) ^119^Sn reconstructed CP-CPMG spectra of 10 wt% Sn-beta zeolite and (c) ^13^C CP spectra of a single-site tungsten TBP metallacycle with microwaves on (top) and off (bottom). All samples were impregnated with a 16 mM solution of **PyPolB-D2[G3]** in TCE. Asterisks mark spinning side bands. NMR acquisition details are given in the ESI.†

In order to assess the effect of the dendrimer in preventing close approach of the free radical to surface sites, well-defined phosphonate-grafted silica nanoparticles (**P@SiO_2_**, Fig. 1a) were impregnated with polarizing solutions containing either non-dendritic or dendritic **bTUreaB** PAs. The surface enhancements (*ε*_^31^P CP_), the proton DNP build-up time (*T*_DNP_), the ^31^P coherence life time (*T*′_2 ^31^P_) and the contribution factors (*θ*_^31^P_)^56^ at the surface were measured. The results, which were recorded on a Bruker 600 MHz (14.1 T) DNP spectrometer, are summarized in Table 3. The enhancements at the surface are roughly the same, which suggests that the **bTUreaB** PAs, dendritic or not, exhibit essentially the same DNP efficiency, as was the case in bulk solutions. Similar *T*_DNP_ (∼2 s) was also observed for all **bTUreaB** PAs. More interestingly, the ^31^P *T*′_2_ increases as we move from **C3** (non-dendritic) to **D1[G3]** ≈ **D1[G4]** to **D2[G3]**, indicating that the free radical centers are further away from the surface as the dendritic structure becomes larger. A similar trend was observed for the ^31^P contribution factor,^56^ for which we observed a significant increase with **D2[G3]**. This suggests that paramagnetic bleaching is reduced at the surface of the material with larger dendritic PA. Thus, **D2[G3]** with 3 dendritic wedges and a hydrodynamic diameter of ∼2.3 nm appears to prevent close approach of the biradical to the surface. In contrast, the results suggest that the **D1[G3]/[G4]** dendrimers may flatten on the surface, leading to a relatively stronger interaction between the free radical and the surface.

**Table 3:** MAS DNP properties of **bTUreaB** PAs impregnated on **P@SiO_2_** ^*a*^

	*ε*_^31^P CP_ ^*b*^	*T*_DNP_ (s)	*T*′_2 ^31^P_ (ms)	*θ*_^31^P_ ^*c*^
**bTUreaB-C3**	7	1.6(1)	6.7(2)	0.43(4)
**bTUreaB-D1[G3]**	9	2.2(1)	7.6(2)	0.42(4)
**bTUreaB-D1[G4]**	9	2.0(1)	7.8(3)	0.42(4)
**bTUreaB-D2[G3]**	8	2.3(1)	8.9(2)	0.51(5)

^*a*^See ESI for more details.

^*b*^The estimated errors are within 2.5% based on the signal-to-noise ratios.

^*c*^ ^31^P contribution factor = [MW OFF with radical]/[MW OFF without radical] per unit mass. See details in ESI.

We then evaluated the DNP SENS efficacy of **PyPolB-D2[G3]** on several representative materials (Fig. 1). First, we impregnated **P@SiO_2_** with a 16 mM solution of **PyPolB-D2[G3]** in TCE. The ^31^P enhancement measured at 600 MHz (14.1 T) was 17 (Fig. 1a). As was the case for bulk solutions, this enhancement is much higher than those obtained with the **bTUreaB** PAs (Table 3). Secondly, we tested **PyPolB-D2[G3]** on a hydrated Sn-beta zeolite^14^ sample by acquiring the ^119^Sn CP spectra with Carr-Purcell Meiboom-Gill (CPMG) echo train acquisition.^57^ The ^119^Sn enhancement was found to be around 4 (Fig. 1b). Notably, given the pore size of the Sn-beta (∼7 Å), **PyPolB-D2[G3]** does not enter the pores. Hence, the DNP enhanced polarization is transferred through proton spin diffusion *via* the TCE molecules and the adsorbed water on the inner-pore surface, which may explain an overall low enhancement.^14,17,32^ Finally, the performance of **PyPolB-D2[G3]** was evaluated on the recently reported metallacyclobutane metathesis intermediates,^16^ and a surface ^13^C enhancement of 8 was obtained. We note that the higher enhancement reported with TEKPol (*ε*_^13^C CP_ ∼ 23, at 14.1 T) originates from the intrinsic different efficiency between PyPol and TEKPol.

### Dendritic PA on a highly reactive heterogeneous catalyst

**Fig. 2:**
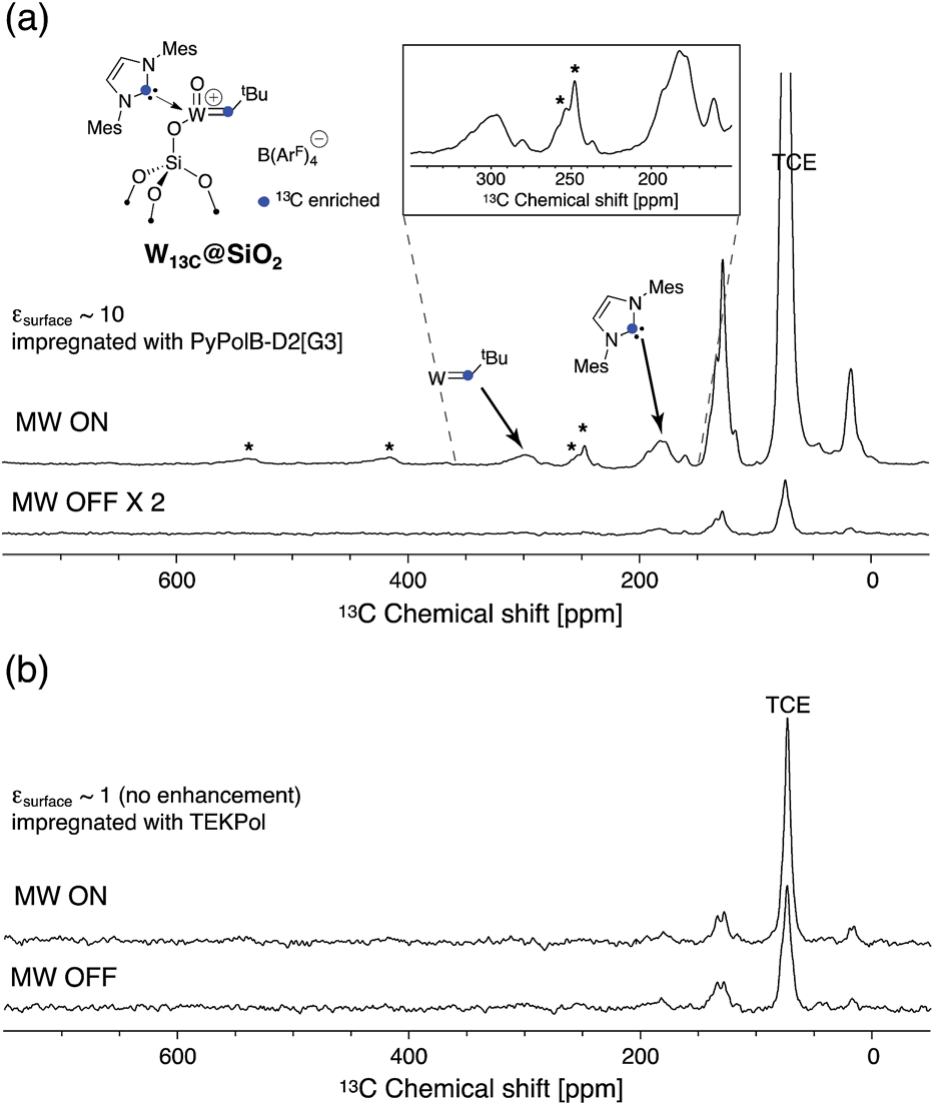
DNP ^13^C CP spectra of **W_^13^C_@SiO_2_** impregnated with (a) 16 mM **PyPolB-D2[G3]** (inset shows the enlarged region between 250 and 350 ppm) and (b) 16 mM TEKPol solutions in TCE with microwaves on (top) and off (bottom) on a 400 MHz (9.4 T) DNP spectrometer. MAS = 12 kHz, recycle delay = (a) 4 s; (b) 4.5 s, contact time = 0.5 ms, number of scans = (a) 2048 (for MW ON), 1024 (for MW OFF); (b) 616. Asterisks mark spinning side bands.

Furthermore, to validate the hypothesis that **PyPolB-D2[G3]** prevents contact of the radical with the surface sites, we studied a highly reactive single-site cationic alkene metathesis heterogeneous catalyst (**W_^13^C_@SiO_2_**),^47^ for which conventional binitroxide radicals do not yield to any DNP effect. This is illustrated in Fig. 2b where no DNP enhancement was observed at the surface when the material was impregnated with a 16 mM TEKPol TCE solution (Fig. 2b). We postulate that TEKPol reacted or coordinated upon contact with the tungsten center. Using a ^13^C-labeled surface complex to speed up the exploration, we observed a ^13^C enhancement of around 10 at the surface (Fig. 2a) for **W_^13^C_@SiO_2_** impregnated with a 16 mM solution of **PyPolB-D2[G3]** in TCE on a Bruker 400 MHz (9.4 T) DNP spectrometer. The results suggest that the dendritic structure largely reduces the interaction between the free radical and the reactive surface site, albeit not fully as evidenced by the observation of ^29^Si NMR signals from the dendritic backbone (Fig. S8a†), whereas we barely observed such signal when the PA was impregnated on pure silica (Fig. S8b†).^58^ Despite a relatively low enhancement (*ε* ∼ 10), under these conditions we could observe all the expected resonances including the isotropic peaks of the alkylidene carbon at 296 ppm and of the N-heterocyclic carbene at 184 ppm after a short period of signal averaging (∼15 minutes) (also see Fig. S9†).^59^ It is worth mentioning that the ^13^C signal of the surface alkylidene is typically very difficult to observe even with ^13^C enrichment, mostly due to its large chemical shift anisotropy (CSA),^60^ usually over 500 ppm, and it typically requires hours or even days of acquisition with conventional solid-state NMR.

## Conclusion

In summary, a series of mono- and bi-nitroxide radicals with bulky hydrophobic carbosilane dendrimers were designed and synthesized. EPR results suggest that there is no significant aggregation between dendrimers in frozen solutions at 100 K. As expected, the dendritic monoradical shows poor DNP enhancement in the bulk solution due to the spatial separation between two nitroxide radicals. On the contrary, the dendritic biradicals are as good PAs as their molecular analogues. Furthermore, the most rigid and bulky molecule **D2[G3]** effectively reduces significant interaction between the free radical and silica surface sites, reducing the fast relaxation of surface substrates induced by the unpaired electrons. More importantly, **PyPolB-D2[G3]** is found to be significantly less reactive and more shielded than TEKPol, making possible the detection here of a highly reactive heterogeneous metathesis catalyst due to its large steric hindrance hampering the interaction between the free radical and the surface site.

## Author contribution

WCL, AL, LE and CC designed research. WCL, TCO, DG and MY performed research. FB and CS provided binitroxide radicals. MP, RS and MS provided analytes. WCL, TCO, DG, MY, MRB, GJ, PT, OO, AL, LE and CC analyzed data. WCL, TCO, AL, LE and CC wrote the paper.
