# Supplementary material for: Dendritic polarizing agents for DNP SENS
Source: Chem Sci. 2016 Aug 22;8(1):416–22. doi: 10.1039/c6sc03139k (PMC5365053; doi:10.1039/c6sc03139k)
Supplement: Supplementary file 1 [file SC-008-C6SC03139K-s001.pdf]

Supporting Information

Dendritic Polarizing Agents for DNP SENS

Wei-Chih Liao,[a] Ta-Chung Ong,[a] David Gajan,[b] Florian Bernada,[c] Claire Sauvée,[c] Maxim Yulikov,[a] Margherita Pucino,[a] Roman Schowner,[d] Martin Schwarzwälder,[a] Michael R. Buchmeiser,[d] Gunnar Jeschke,[a] Paul Tordo,[c] Olivier Ouari,[c] Anne Lesage,[b] Lyndon Emsley[e] and Christophe Copéret*[a]

Table of contents

I. Hydrodynamic diameters of the dendrimers S3
Table S1. Diffusion coefficients and hydrodynamic diameters of the dendrimers S3
Figure S1. Structures of the corresponding non-dendritic PAs S3

II. Electronic paramagnetic resonance spectroscopy S4
a. Continuous wave (CW) EPR S4
Figure S2. X band CW EPR spectra S5
b. Pulse EPR spectroscopy S5
Figure S3. Inversion recovery pulse sequence in pulse EPR to determine T_{1e} S5
Figure S4. T_{1e} relaxation traces (*blue*) and fits (*red*) S6
Figure S5. Hahn echo pulse sequence in pulse EPR to determine T_{2e} S7
Figure S6. T_{2e} relaxation traces (*blue*) and fits (*red*) S7

III. DNP enhanced NMR spectroscopy S8
a. General description S8
b. Bulk solution enhancement S8
Figure S7. DNP enhanced 13C CP spectra of a) 16 mM **bTUreaB-D1[G4]** and b) 16 mM **PyPolB-D2[G3]** bulk solutions. S9
c. DNP performances of dendritic biradiclas on materials S9
d. DNP properties of bTUreaB series on P@SiO$_2$ S10
e. DNP on W$_{13C}$@SiO$_2$ S11
Figure S8. DNP enhanced ^{29}Si CP-CPMG spectra S12
Figure S9. DNP enhanced ^{13}C CP spectrum **of W$_{13C}$@SiO$_2$** S13
Figure S10. DNP enhanced ^{13}C CP spectrum **of W$_{13C}$@SiO$_2$** S14

IV. Experimental section of synthesis S15
a. General description S15
b. Synthesis and characterization S16
Table S2. Experimental details of all ester bond coupling reactions S16
Table S3. Experimental details of all amide bond coupling reactions S18

V. References S45

I. Hydrodynamic diameters of the dendrimers

The hydrodynamic diameters of the dendrimers were estimated by measuring their diffusion coefficients in dilute $CDCl_3$ solution with diffusion-ordered spectroscopy (DOSY). Measurements were done on a Bruker 400MHz NMR spectrometer with a standard 5 mm probe for solution samples. The temperature of the sample was kept at 25 ^oC to avoid solution convection. 64 increments were collected for each experiment. The intensity decay curves were optimized to be within 95% and 5% region depending on the ^1H T_1 relaxation, and were fit with the topspin T_1/T_2 fitting module to afford the corresponding diffusion coefficients. Hydrodynamic diameters were calculated based on the Stokes-Einstein equation.

$$\boldsymbol{D} = \frac{kT}{6\pi\eta r}$$

D: diffusion coefficient; *k*: Boltzmann constant; *T*: temperature; η: the viscosity of the liquid, $CDCl_3$ (0.539×10^{-3} $kgs^{-1}m^{-1}$ in this case)[1]; *r*: the hydrodynamic radius of the molecule.

Table S1. Diffusion coefficients and hydrodynamic diameters of the dendrimers

	D (m^2s^{-1})	Hydrodynamic diameter (nm)
**TEMPO-D1[G3]$_{CH3}$[a]** (quenched by ascorbic acid)	4.8×10^{-10}	1.7
**D1[G4]OBn**	3.6×10^{-10}	2.3
**D2[G3]**	3.5×10^{-10}	2.3

[a] CH_3 as end groups of the dendritic structure.

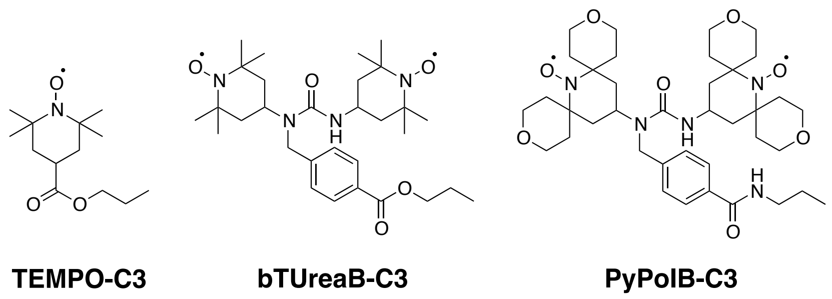

Figure S1. Structures of the corresponding non-dendritic PAs

II. Electronic paramagnetic resonance spectroscopy

a. Continuous wave (CW) EPR

Continuous Wave (CW) EPR spectra were recorded on a Bruker EMX X Band spectrometer (9.5 GHz microwave frequency). The conversion time was set to 40.96 ms and the time constant to 5.12 ms. 1024 data points were recorded. The modulation frequency was 100 kHz and the modulation amplitude was 1 G. In all measurements, attenuation was varied such that no saturation was observed.

Spin count

The samples were filled in Hirschmann glass capillaries, which were then closed with putty. The sample position in the cavity was carefully optimized. The spectra were recorded at room temperature with a sweep width of 600 G and an attenuation of 26 dB. The amount of radical was determined by double integration of the CW spectra and by referencing to a calibration curve of TEMPO solutions in TCE measured for the concentration range between 0.4 and 80 mM. Data was processed with MATLAB® (R2011a, MathWorks Inc.).

Peak-to-peak line width

Each sample was prepared as a 30 mM TCE solution and filled in a 3.0 mm quartz tube. All spectra were recorded at 110 K using a nitrogen flow cryostat. Attenuation was 40 dB. The EPR spectrum of a nitroxide radical consists of three lines due to strong hyperfine interaction with the ^{14}N nucleus. For the line width measurements we have used the central line, which is the least broadened by the g-tensor and hyperfine anisotropies and which therefore is the most sensitive to the dipolar broadening. For the obtained signal-to-noise ratios, the estimated line width errors were within 5 %.

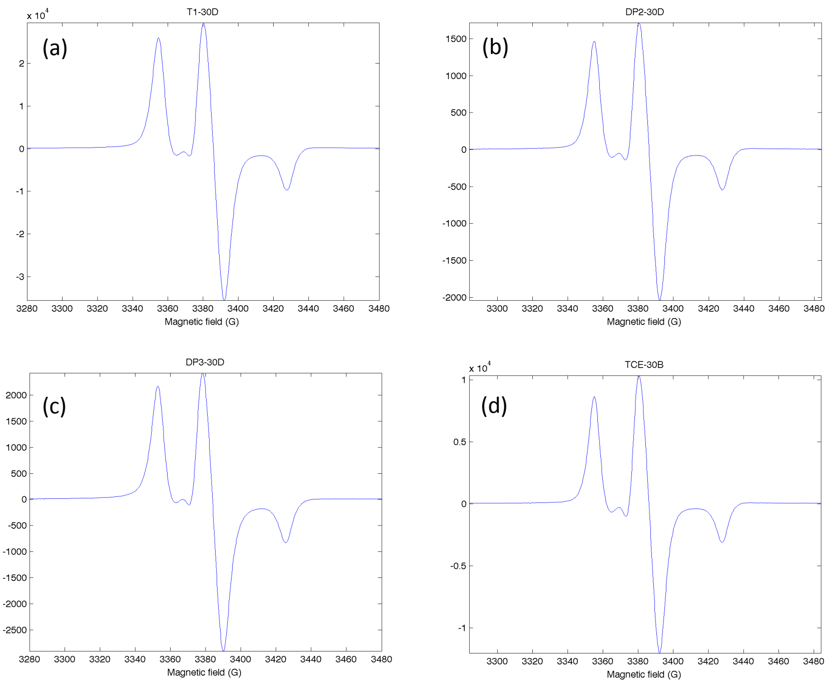

Figure S2. X band CW EPR spectra of (a) **TEMPO-C3**, (b) **TEMPO-D1[G3]**, (c) **TEMPO-D2[G4]** and (d) **TEMPO-D2[G3]** as 30 mM TCE solutions at 100K.

b. Pulse EPR spectroscopy

All pulse experiments were recorded at W band (94 GHz) on a Bruker Elexsys E680 EPR spectrometer. The temperature was stabilized with an Oxford helium flow cryostat and was held at 100 K for all experiments. All samples were prepared as 16 mM TCE solutions to be close to actual DNP conditions.

T_{1e} Measurements

The longitudinal relaxation time T_{1e} was determined by performing an inversion recovery experiment. The used pulse sequence is shown in Figure S3. Pulse lengths used were 80 ns for the inversion pulse, 40 ns for the π/2 and 80 ns for the refocusing π pulse, respectively. The initial d_{var} was 1000 ns, and the time increment was 500 ns. Error bars are estimated to be approximately 5 % as good signal-to-noise traces were recorded for all the samples.

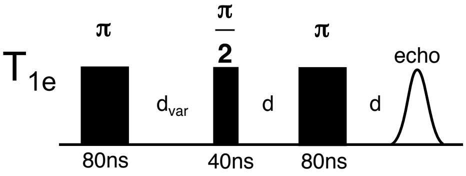

Figure S3. Inversion recovery pulse sequence in pulse EPR to determine T_{1e}

The inversion recovery time traces were fit using a stretched exponential function (see below) and the first moment of the distribution is discussed in the text as a mean relaxation time.

$$I(t) = I_0 + I_1 \cdot e^{-\left(\frac{t}{T_{1e}^*}\right)^{\beta}}$$

$$\langle T_{1e} \rangle \equiv \int_0^{\infty} e^{-(x/T_{1e}^*)^{\beta}} dt = \frac{T_{1e}^*}{\beta} \Gamma(\frac{1}{\beta})$$

Stretched exponential function was used for fitting of the inversion recovery traces on top and the function to determine the mean relaxation time <T_{1e}> on the bottom. I_0 is the initial intensity, I_1 the proportionality factor, T_{1e}^* the decay time parameter and β the stretching parameter. The mean relaxation time <T_{1e}> discussed in the text is the first moment of the distribution and is determined using the decay time parameter T_{1e}^* and the stretching parameter β.

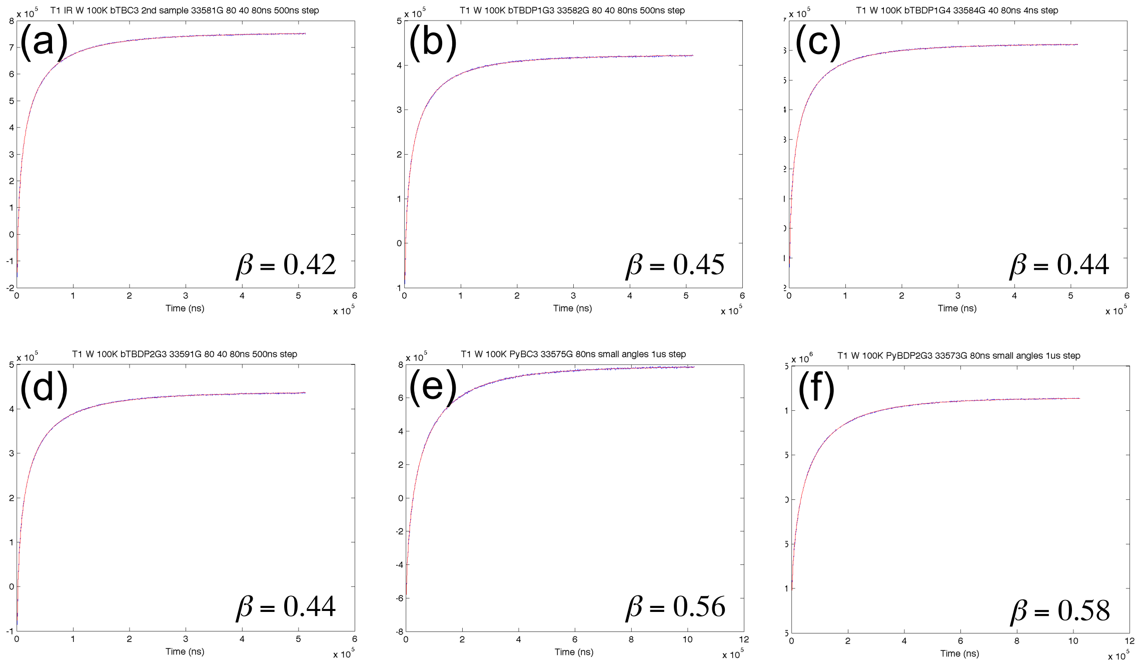

Figure S4. T_{1e} relaxation traces (*blue*) and fits (*red*) of **(a) bTUreaB-C3**, **(b) bTUreaB-D1[G3]**, **(c) bTUreaB-D1[G4]**, **(d) bTUreaB-D2[G3]**, **(e) PyPolB-C3** and **(f) PyPolB-D2[G3]**. The corresponding stretching parameters (β) are indicated.

T_{2e} Measurements

The transverse relaxation time T_{2e} was determined using a variable-delay Hahn echo pulse sequence. The used pulse sequence is shown in Figure S4. Pulse lengths of

40 ns for the π/2 and 80 ns for the refocusing π pulse were used. The initial d_{var} was at 400 ns, the time increment was 4 ns. The two-pulse echo decay traces were fitted using a monoexponential function. Error bars are estimated to be approximately 5 % as good signal-to-noise traces were recorded for all the samples.

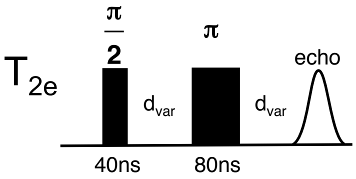

Figure S5. Hahn echo pulse sequence in pulse EPR to determine T_{2e}

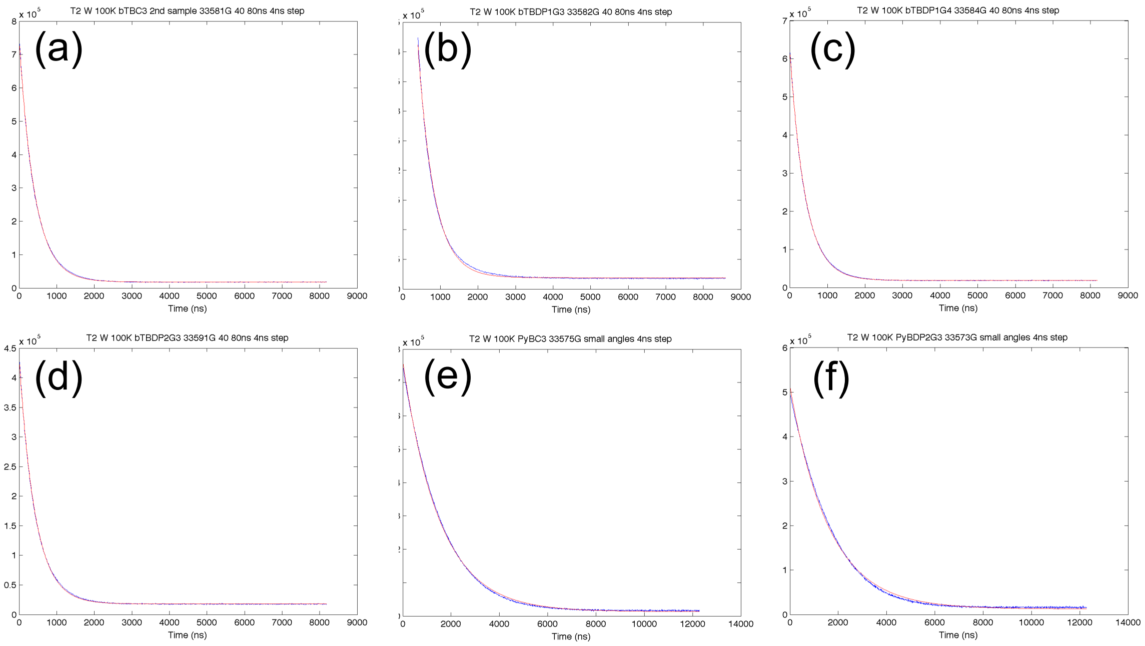

Figure S6. T_{2e} relaxation traces (*blue*) and fits (*red*) of **(a) bTUreaB-C3**, **(b) bTUreaB-D1[G3]**, **(c) bTUreaB-D1[G4]**, **(d) bTUreaB-D2[G3]**, **(e) PyPolB-C3** and **(f) PyPolB-D2[G3]**.

III. DNP enhanced NMR spectroscopy

a. General description

All DNP enhanced solid-state NMR experiments were either conducted on a Bruker 600 MHz (14.1 T) spectrometer using a 3.2 mm HX or HXY probe located at ETH Zürich, or on a Bruker 400 MHz (9.4 T) spectrometer using a 3.2 mm HXY probe located at the Centre de RMN à Trés Hauts Champs at ISA Lyon. All samples were cooled to 100 K by a cryogenic heat exchanger system. Microwaves used to drive the DNP Cross Effect are provided by gyrotrons emitting at 263 GHz (400 MHz spectrometer) and 395 GHz (600 MHz spectrometer) with power between 6 to 10 W. The magnetic fields were adjusted by referencing the higher frequency peak of adamantane to 38.5 ppm. TCE peak was used as a second reference for ^{13}C spectra.

For moisture or air sensitive materials, samples were prepared in an argon-filled glove box and inserted in the cryogenic probe within a short period of time (~ 5 min). The dendritic PAs and TEKPol used were dried under high vacuum (10^{-4} mbar) for 16 hours and 5 hours, respectively. The solvent used, 1,1,2,2-tetrachloroethane (TCE), was dried over calcium hydride overnight, then vacuum transferred to a rotaflo flask. All materials were stored in the argon-filled glove box after removing residual moistures and degassing.

b. Bulk solution enhancement

The monoradical was prepared as a 30 mM TCE solution; the biradicals were prepared as 16 mM solutions. 23 μL of the prepared solution were pipetted into a 3.2 mm sapphire rotor, which was then sealed with a siloxane insert to avoid solution leaking. Each sample was deoxygenated by a series of freeze-thaw cycles, *i.e.* insert-eject cycles, inside the cryogenic DNP probe to reach the maximum DNP enhancement.

Ramped cross polarization (CP) from ^{1}H to ^{13}C was used for all experiments with contact time between 0.5 to 2.0 ms. SPINAL64[2] was used for ^{1}H decoupling at γB_1 of 100 kHz. The enhancements were measured by comparing the intensity of the ^{13}C spectrum when microwaves were on to the intensity of the ^{13}C spectrum when microwaves were off. The ^{1}H T_1 was measured by saturation recovery experiments. All experiments were carried out with MAS frequency set to 8kHz.

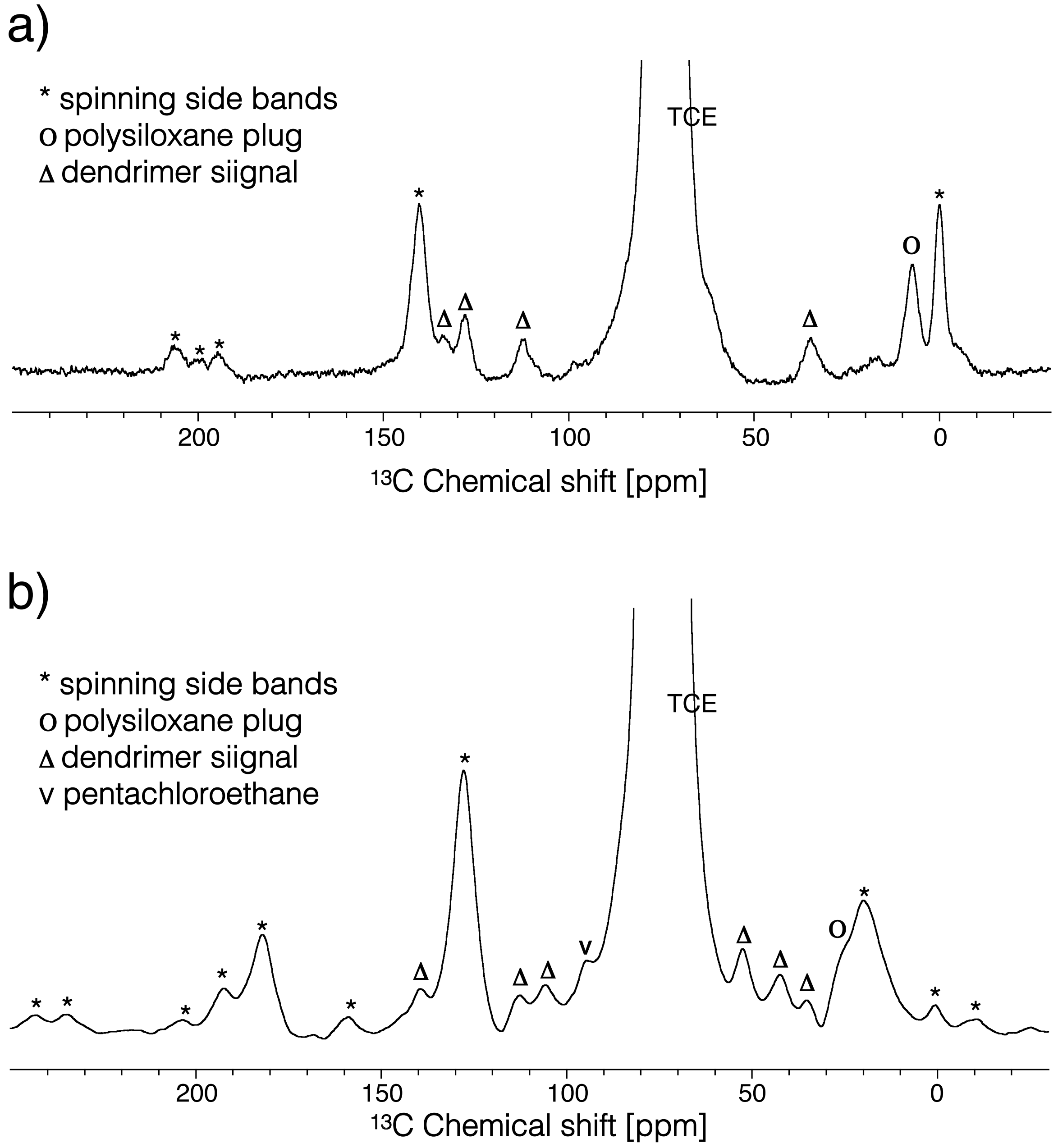

Figure S7. DNP enhanced 13C CP spectra of a) 16 mM **bTUreaB-D1[G4]** and b) 16 mM **PyPolB-D2[G3]** bulk solutions. Number of scans = a) 2048, b) 1616. D1 = a) 5 s, b) 2 s. Contact time = a) 2 ms, b) 0.6 ms.

c. DNP performances of dendritic biradiclas on materials

All samples were measured on a Bruker 600 MHz (14.1 T) DNP spectrometer.

P@SiO$_2$ (moisture sensitive) was incipient wetness impregnated with 16 mM **PyPolB-D2[G3]** TCE solution. ^{1}H-^{31}P CP Hahn-echo experiments were performed to acquire the ^{31}P NMR spectra with the MAS frequency set to 10 kHz. Ramp CP from ^{1}H to

^{31}P was used for all experiments with a contact time of 1.5 ms. SPINAL64[2] was used for ^{1}H decoupling at γB_1 of 100 kHz. ^{1}H T_1 was measured by saturation-recovery experiment, and the recycle delay was set to be 5 x T_1 as 30 s (for the experiments described in SI *III.d*). The enhancement was measured by comparing the intensity of the ^{31}P spectrum when microwaves were on to the intensity of the ^{31}P spectrum when microwaves were off.

10 wt.-% Sn-beta zeolite was incipient wetness impregnated with 16 mM **PyPolB-D2[G3]** TCE solution. ^{1}H-^{119}Sn CP-CPMG experiments were performed to acquire the spikelet ^{119}Sn NMR spectra with the MAS frequency set to 10 kHz. 20 echoes were collected for each experiment. Ramp CP from ^{1}H to ^{119}Sn was used for all experiments with a contact time of 1.0 ms. SPINAL64[2] was used for ^{1}H decoupling at γB_1 of 100 kHz. ^{1}H T_1 was measured by saturation-recovery experiment, and the recycle delay was set to be 1.3 x T_1 as 3.2 s. The enhancement was measured by comparing the intensity of the ^{119}Sn spectrum when microwaves were on to the intensity of the ^{119}Sn spectrum when microwaves were off.

TBP (moisture sensitive) was incipient wetness impregnated with 16 mM **PyPolB-D2[G3]** TCE solution. ^{1}H-^{31}C CP experiments were performed to acquire the ^{13}C NMR spectra with the MAS frequency set to 10 kHz. Ramp CP from ^{1}H to ^{31}C was used for all experiments with a contact time of 2.0 ms. SPINAL64[2] was used for ^{1}H decoupling at γB_1 of 100 kHz. ^{1}H T_1 was measured by saturation-recovery experiment, and the recycle delay was set to be 1.3 x T_1 as 4.5 s. The enhancement was measured by comparing the intensity of the ^{13}C spectrum when microwaves were on to the intensity of the ^{13}C spectrum when microwaves were off.

d. DNP properties of bTUreaB series on P@SiO$_2$

P@SiO$_2$ (moisture sensitive) was separately impregnated with 16 mM **bTUreaB**-PAs TCE solutions. Each sample was prepared with the ratio of 1 mg of solid to 1μL of 16mM biradical solution, and the exact masses used were recorded.

All measurements were done on a Bruker 600 MHz DNP spectrometer with the MAS frequency set to be 10 kHz. ^{1}H-^{31}P CP Hahn-echo experiments were performed to acquire the ^{31}P NMR spectra. Ramp CP from ^{1}H to ^{31}P was used for all experiments with a contact time of 1.5 ms. SPINAL64[2] was used for ^{1}H decoupling at γB_1 of 100 kHz. ^{1}H T_1 was measured by saturation-recovery experiment. ^{31}P T_2 was measured by varied-delay CP Hahn-echo experiments. The enhancement was measured by comparing the intensity of the ^{31}P spectrum when microwaves were on to the intensity of the ^{31}P spectrum when microwaves were off. The contribution factor measurements were

adjusted from the procedure described previously.[3] The signal integral of the sample impregnated with the biradical solution with microwave off was compared to the signal integral of the sample with pure TCE impregnation. The integral was normalized by mass and by number of scans. The recycle delay was set to be 5 x T_1 in order to complete full relaxation. Accordingly the value of 1-θ represents the fraction that does not contribute to the observable signal.

e. DNP on W_{13C}@SiO_2

W_{13C}@SiO_2 (moisture and air sensitive) was separately impregnated with 16 mM **PyPolB-D2[G3]** and TEKPol TCE solutions. All measurements were done on a Bruker 400 MHz DNP spectrometer. The MAS frequency was set to be 12 kHz or 10 kHz for 1D experiments and 5 kHz for the CPMAT experiment. Ramp CP from ^{1}H to ^{13}C was used for all ^{13}C CP experiments with a contact time of 0.5 ms. Ramp CP from ^{1}H to ^{29}Si was used for all ^{29}Si CP-CPMG experiments with a contact time of 7.0 ms, and 30 echoes were collected for each experiment. SPINAL64[2] was used for ^{1}H decoupling at γB_1 of 100 kHz. ^{1}H T_1 was measured by a saturation-recovery experiment, and the recycle delay was set to be 1.3 x T_1. The enhancement was measured by comparing the intensity of the spectrum (^{13}C or ^{29}Si) when microwaves were on to the intensity of the spectrum (^{13}C or ^{29}Si) when microwaves were off.

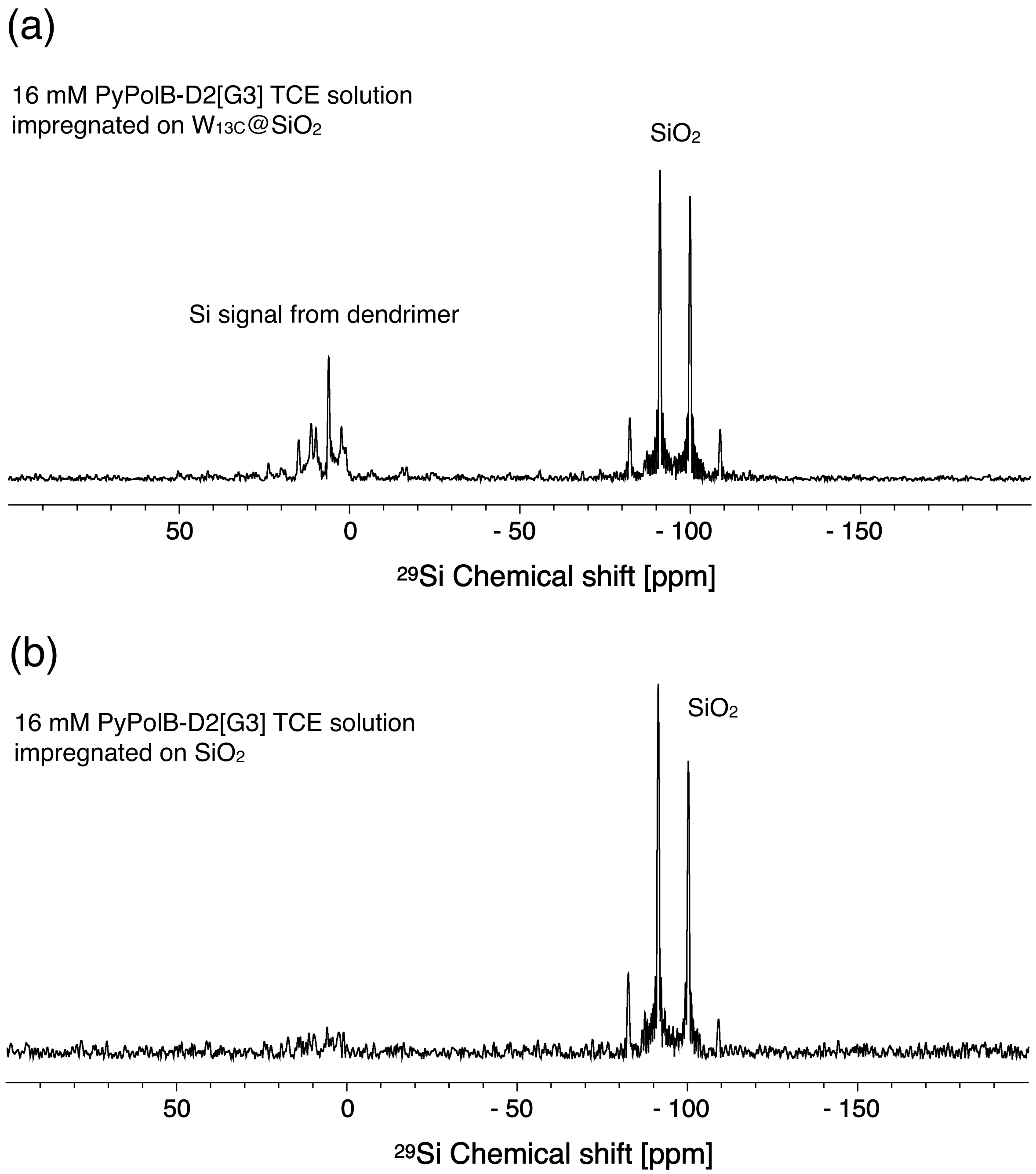

Figure S8. DNP enhanced ^{29}Si CP-CPMG spectra of **(a) W_{13C}@SiO_2** and **(b)** SiO_2* impregnated with 16 mM **PyPolB-D2[G3]** TCE solution on a Bruker 400MHz DNP spectrometer.

*Partially dehydroxylated silica (moisture sensitive) was impregnated with 16 mM **PyPolB-D2[G3]** TCE solution.

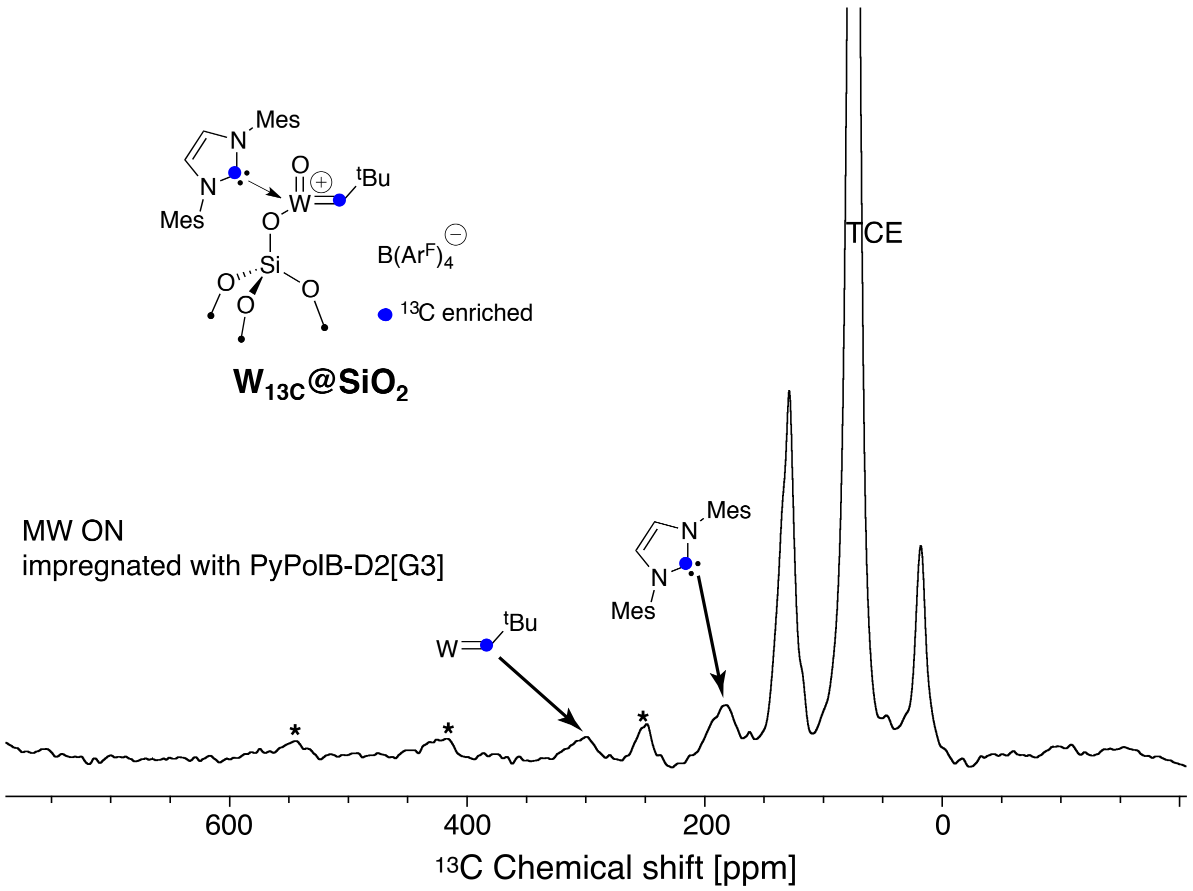

Figure S9. DNP enhanced ^{13}C CP spectrum of **W_{13C}@SiO_2** impregnated with 16 mM **PyPolB-D2[G3]** TCE solution on a Bruker 400MHz DNP spectrometer. D1 = 3.7 s; number of scans = 224; overall experimental time ~ 15 min. Signal-to-noise ratio = 241 calculated based the alkylidene carbon peak. Asterisks mark spinning side bands.

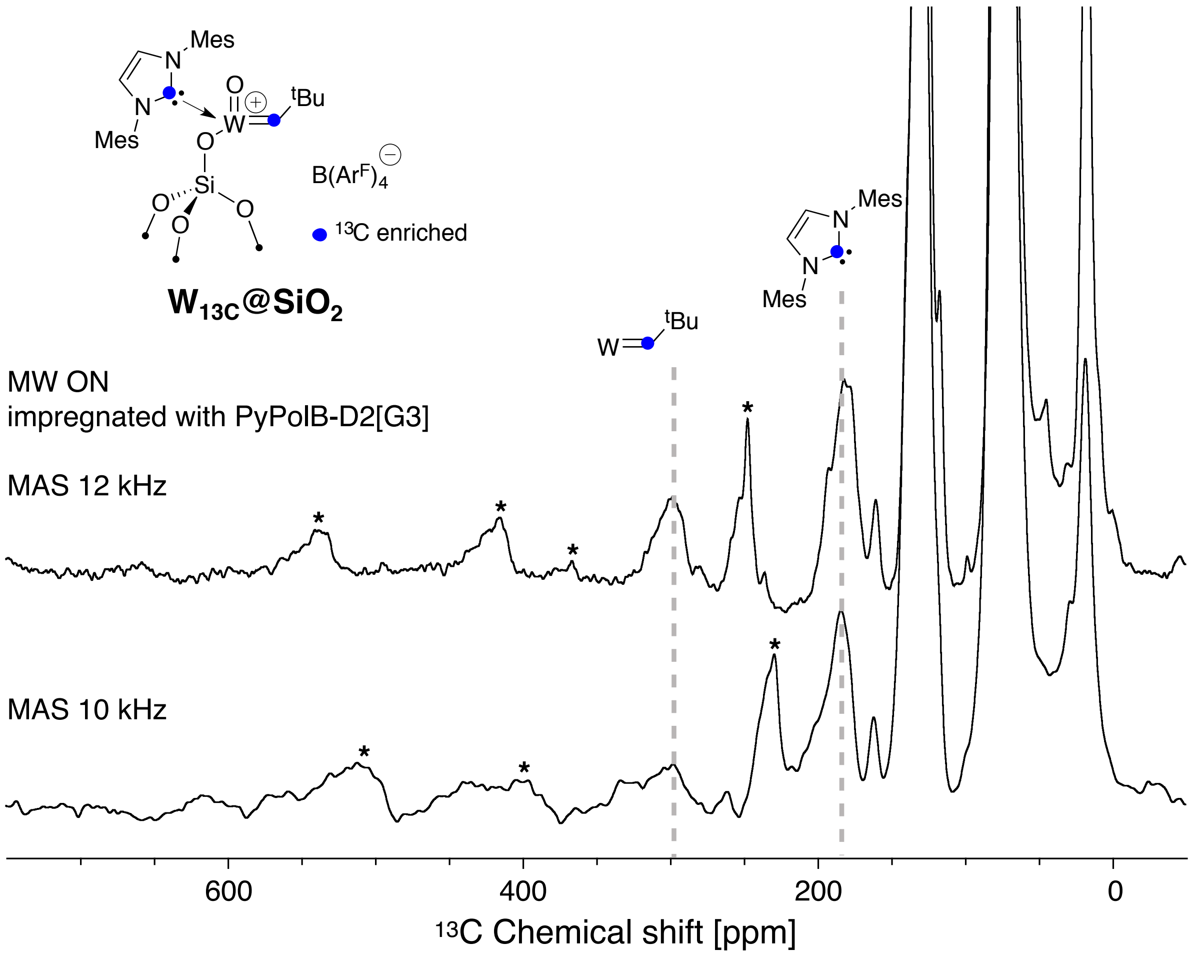

Figure S10. DNP enhanced ^{13}C CP spectrum of **W_{13C}@SiO$_2$** impregnated with 16 mM PyPolB-D2[G3] TCE solution on a Bruker 400MHz DNP spectrometer with MAS at 12 kHz (*top*) and 10 kHz (*bottom*). Asterisks mark spinning side bands.

IV. Experimental section of synthesis

a. General information

For reactions carried out under N_2, a standard N_2–filled Schlenk line was used. Other reactions were performed in atmospheric environment. Dry dichloromethane (DCM), diethyl ether (Et_2O) and toluene were purified by passage through double solvent purification alumina columns (MBraun). Dry tetrahydrofuran (THF) was dried over sodium with benzophenone as indicator, and further vacuum transferred and stored in a rotaflo flask. Dry dimethylformamide (DMF) was purchased from Sigma-Aldrich (99.8% extra dry over Molecular Sieve) and used as received. Other solvents: Pentane, cyclohexane, Et_2O and ethyl acetate (EA) were purchased from the ETH HCI-shop Tanklager and used as received. DCM and THF were purchased from Acros and used as received.

Solution NMR measurements were done on Bruker DPX-300 MHz or 400 MHz or 500 MHz NMR spectrometers (specified in the following text) using $CDCl_3$ as a solvent. The ^1H and ^{13}C chemical shifts are referenced to the chloroform peak (7.26 ppm for ^1H and 77.00 ppm for ^{13}C). High-resolution mass spectra were measured by the MS-service of the Laboratorium für Organische Chemie, ETH Zürich and by Aix-Marseille Université Mass Spectrum Facility, Spectropole Saint Jérôme Marseille. Values are given as *m/z*.

Allylbenzyl ether (Sigma-Aldrich and TCI), platinum(0)-1,3-divinyl-1,1,3,3-tetra-methyldisiloxane complex solution (Karsted't's catalyst, Sigma-Aldrich), allylmagnesium chloride solution (Sigma-Aldrich), 2,3-dichloro-5,6-dicyano-1,4-benzoquinone (Sigma-Aldrich), trichloro(3-chloropropyl)silane (ABCR), dichlorophenylsilane (ABCR), trichlorosilane (Sigma-Aldrich), benzylmagnesium chloride solution (Sigma-Aldrich), sodium azide (Sigma-Aldrich), sodium iodide (Sigma-Aldrich), triphenylphosphine (Sigma-Aldrich), methyl-d3-magnesium iodide solution (Sigma-Aldrich), chloroplatinic acid hexahydrate (Strem), tetrabutylammonium hydrogensulfate (Sigma-Aldrich), dimethylaminopyridine (Sigma-Aldrich), 1-(3-dimethylaminopropyl)-3-ethylcarbodiimide hydrochloride (Sigma-Aldrich), N,N'-dicyclohexylcarbodiimide (Sigma-Aldrich), 1-hydroxybenzotriazole (Sigma-Aldrich) and diisopropylamine (Sigma-Aldrich) were purchased and used as received.

The chloroplatinic acid hexahydrate DCM solution (1 equiv.) was mixed with the tetrabutylammonium hydrogensulfate water solution (2 equiv.) for 3 hours. The organic layer was separated from the aqueous layer and dried over magnesium sulfate. The solvent was removed *in vacuo* and afforded an orange solid. The orange solid was used as bis(tetrabutylammonium) hexachloroplatinate (Lukevics' catalyst) in the following reaction directly without further purification.

b. Synthesis and characterization

Ester bond coupling

The ester coupling reagent (DCC or EDCI HCl salt, 1.1 equiv., Table S4) and DMAP (catalytic amount, approximately 10 mol%) were added into a solution of a OH-functionalized substrate (**D1[G3]** or **D1[G4]**, 1 equiv., or propyl alcohol, 2.5 equiv., the amount of material used, W_{OH} is specified in Table S4.) and a COOH-functionalized substrate (4-carboxy TEMPO or **bTUreaB**, 1 equiv., the amount of material used, W_{COOH} is specified in Table S4.) in dichloromethane (2 mL). After the mixture was stirred for 16 hours at room temperature, it was poured into water and extracted with dichloromethane (50 mL x 3). The organic layers were collected, washed with water (50 mL x 3) and brine (50 mL) and dried over sodium sulfate. The solvent was removed in *vacuo*. The residue was purified by preparative thin layer chromatography, and the corresponding eluent was cyclohexane/EA (15/1 for TEMPO-PAs, and 2/1 for **bTUreaB**-PAs). The final product was extracted by stirring the product-adsorbed silica gel in EA (100 mL). After stirring for 10 min at room temperature, the slurry was filtered, and the solvent was removed *in vacuo*. The final residue was re-dissolved in dichloromethane (less than 5 mL) and filtered through a neutral cellulose syringe filter to remove residual silica gel. The solvent was removed *in vacuo* to afford the final product (red solids for **TEMPO-C3** and **bTUreaB-C3**; red sticky oils for **TEMPO-D1[G3]/[G4]** and **bTUreaB-D1[G3]/[G4]**).

Table S2. Experimental details of all ester bond coupling reactions

product	W_{OH} (mg)	W_{COOH} (mg)	DCC (mg)	EDCI[a] (mg)	Yield (%)
**TEMPO-C3**	90[b]	100	149	-	72
**TEMPO-D1[G3]**	275	44	45	-	87
**TEMPO-D1[G4]**	225	20	21	-	87
**bTUreaB-C3**	19[b]	25	-	14	88
**bTUreaB-D1[G3]**	63	27	-	11	48
**bTUreaB-D1[G4]**	97	19	-	9	77

[a] EDCI HCl salt. [b] Unit in μL.

TEMPO-C3: HRMS (MALDI) *m/z* = 242.1751 [$C_{13}H_{24}NO_3$]; EPR spin count = 29.1 mM for a 30 mM TCE solution.

TEMPO-D1[G3]: HRMS (MALDI) *m/z* = 1411.8616 [$C_{81}H_{94}D_{24}NO_3Si_7 + K^+$]; EPR spin count = 30.9 mM for a 30 mM TCE solution.

TEMPO-D1[G4]: HRMS (MALDI) *m*/*z* = 2800.6473 [$C_{161}H_{182}D_{48}NO_3Si_{15}$ + Ag^+]; EPR spin count = 28.9 mM for a 30 mM TCE solution.

bTUreaB-C3: HRMS (MALDI) *m*/*z* = 544.3619 [$C_{30}H_{48}N_4O_5$]; EPR spin count = 15.2 mM for a 16 mM TCE solution.

bTUreaB-D1[G3]: HRMS (MALDI) *m*/*z* = 1676.0944 [$C_{98}H_{119}D_{24}N_4O_5Si_7$]; EPR spin count = 15.0 mM for a 16 mM TCE solution.

bTUreaB-D1[G4]: HRMS (MALDI) *m*/*z* = 3102.8342 [$C_{178}H_{206}D_{48}N_4O_5Si_{15}$ + Ag^+]; EPR spin count = 16.9 mM for a 16 mM TCE solution.

Amide bond coupling

The amide coupling reagent (EDCI HCl salt, 1.5 equiv., Table S5), HOBt (catalytic amount, approximately 10 mol%) and diisopropylamine (1 equiv., Table S5) were added into a solution of **D2[G3]** (1.1 equiv) or propyl amine (3 equiv.) (the amount of material used, W_{NH2} is specified in Table S5) and a COOH-functionalized substrate (4-carboxy TEMPO or **bTUreaB** or **PyPolB** (1 equiv.) (the amount of material used, W_{COOH} is specified in Table S5) in dichloromethane (2 mL). After the mixture was stirred for 16 hours at room temperature, it was poured into water and extracted with dichloromethane (50 mL x 3). The organic layers were collected, washed with water (50 mL x 3) and brine (50 mL) and dried over sodium sulfate. The solvent was removed *in vacuo*. The residue was purified by preparative thin layer chromatography, using pentane/EA (1/5 for **TEMPO-D2[G3]** and 1/2 for **bTUreaB-D2[G3]**), pure EA for **PyPolB-D2[G3]**, or DCM/methanol (10/1) for **PyPolB-C3** as eluents. The final product was extracted by stirring the product-adsorbed silica gel in EA (100 mL). After stirring for 10 min at room temperature, the slurry was filtered, and the solvent was removed *in vacuo*. The final residue was re-dissolved in dichloromethane (less than 5 mL) and filtered through a neutral cellulose syringe filter to remove residual silica gel. The solvent was removed *in vacuo* to afford the final product (red sticky oils for dendritic PAs; red solid for **PyPolB-C3**).

Table S3. Experimental details of all amide bond coupling reactions

product	W_{NH2} (mg)	W_{COOH} (mg)	EDCI[a] (mg)	DIEA (μL)	Yield (%)
**TEMPO- D2[G3]**	101	6.6	8.6	5.2	70
**bTUreaB-D2[G3]**	81	12	7.0	6.3	81
**PyPolB-D2[G3]**	115	21	9.8	5.4	49
**PyPolB-C3**	8.2[b]	22	7.0	-	82

[a] EDCI HCl salt. [b] Unit in μL.

TEMPO-D2[G3]: HRMS (MALDI) m/z = 3673.8307 [$C_{238}H_{285}N_2O_2Si_{13}$ + Ag^+]; EPR spin count = 28.4 mM for a 30 mM TCE solution.

bTUreaB-D2[G3]: HRMS (MALDI) m/z = 3779.0655 [$C_{255}H_{309}N_5O_4Si_{13}$ – C_7H_6]; EPR spin count = 14.8 mM for a 16 mM TCE solution.

PyPolB-D2[G3]: HRMS (MALDI) m/z = 3946.0999 [$C_{263}H_{317}N_5O_8Si_{13}$ – C_7H_7]; EPR spin count = 15.7 mM for a 16 mM TCE solution.

PyPolB-C3: HRMS (MALDI) m/z = 734.4099 [$C_{38}H_{57}N_5O_8$ + Na^+]; EPR spin count = 13.6 mM for a 16 mM TCE solution.

Synthesis of solid materials

P@SiO_2

0.4 ml (1.92 mmol, 7 equiv.) of diethyl benzylphosphonate dissolved in 6 ml pentane was grafted on 1007 mg (0.26 mmol OH) SiO_2 dehydroxylated at 700 °C.
EA Diethyl benzylphosphonate on $SiO_{2\text{-}700}$: Found: C, 7.61; H, 0.96; P, 1.74 wt%.

10 wt% Sn-beta zeolite, single-site tungsten TBP metallacycle and W_{13C}@SiO_2

These samples were prepared as reported.[4-6]

Synthesis of the binitroxide radicals

bTUreaB

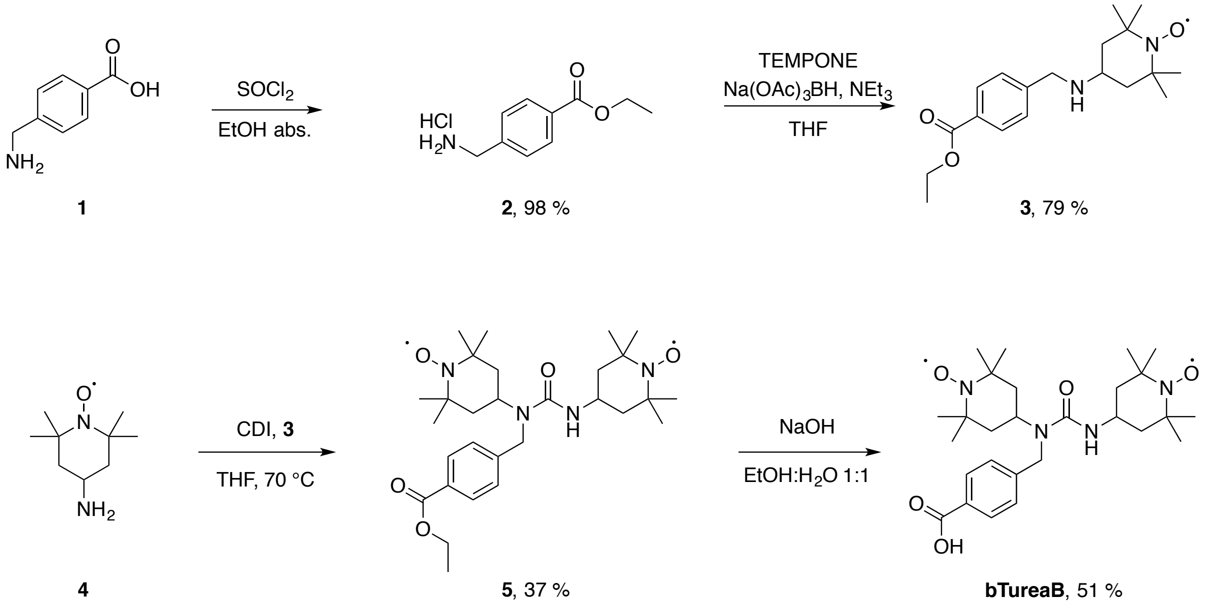

- Ethyl 4-(aminomethyl)benzoate hydrochloride (**2**)

To a solution of 4-(aminomethyl)benzoic acid (10 g, 66 mmol) in absolute EtOH (150 ml) was added dropwise $SOCl_2$ (15 ml, 196 mmol). The reaction was stirred at 70 °C for 5 h. The reaction mixture was concentrated under reduced pressure and Et_2O was added. The precipitate was filtered off to yield ester **2** (14.05 g, 65 mmol, 98 %). ^{1}H NMR (300 MHz, D_2O) : δ 1.38 (t, J=7.15 Hz, 3 H) 4.26 (s, 2 H) 4.41 (m, J=7.15 Hz, 2 H) 7.56 (d, J=8.16 Hz, 2 H) 8.08 (d, J=8.25 Hz, 2 H). ^{13}C NMR (75 MHz, D_2O) : δ 13.39 42.64 62.42 128.87 130.05 130.40 137.84 168.36.

-(4-{[4-(Ethoxycarbonyl)benzyl]amino}-2,2,6,6-tetramethylpiperidin- 1-yl)oxidanyl (**3**)

To a solution of TEMPONE (1.2 equiv) and amine hydrochloride salt **2** (1 equiv) in dry THF was added NEt_3 (1 equiv) and the solution was stirred at room temperature for 2 h. Then, $Na(OAc)_3BH$ (1.6 equiv) was added portionwise and the reaction was stirred at room temperature for 16 h. After this time, the mixture was concentrated under reduced pressure. The residue was solubilized in CH_2Cl_2, washed with a saturated aqueous solution of $NaHCO_3$, dried on Na_2SO_4, concentrated under reduced pressure and the crude product was purified by SiO_2 column chromatography with CH_2Cl_2/MeOH (9/1) to give desired compound **3** (79 %). ^{1}H NMR (300 MHz, $CDCl_3$ + phenylhydrazine) : δ ppm 1.02 (s, 6 H) 1.11 (s, 6 H) 1.23-1.35 (m, 5 H) 1.71 - 1.85 (m, 2 H) 2.80 (tt, J=11.71, 3.42 Hz, 1 H) 3.78 (s, 2 H) 4.28 (q, J=7.12 Hz, 2 H) 7.33 (d, J=8.07 Hz, 2 H) 7.92 (d, J=8.07 Hz, 2 H). HRMS-ESI: m/z calcd for $C_{19}H_{30}N_2O_3$ $[M+H]^+$ 334.2251, found 334.2250.

-Ethyl 4-{[1-(1-oxyl-2,2,6,6-tetramethylpiperidin-4-yl)][(1-oxyl-2,2,6,6tetramethylpiperi-din-4-yl) carbamoyl] amino]methyl}benzoate (**5**)

Under argon, to a solution of 1,1'-carbonyldiimidazole (CDI) (1 equiv) in THF was added a solution of 4-aminoTEMPO (1 equiv) in THF at 0 °C. The solution was stirred at 30 min at room temperature. Then a solution of secondary amine **3** (1.1 equiv) was added and the resulting mixture was stirred at 70 °C for 6 h. The solution was concentrated under reduced pressure and the residue was solubilized in CH_2Cl_2 and washed with a saturated aqueous solution of $NaHCO_2$. The organic layer was dried on Na_2SO_4, concentrated under reduced pressure and the crude product was purified by SiO_2 column chromatography with CH_2Cl_2/MeOH to give the desired compound **5**(37 %).

HRMS-ESI: m/z calcd for $C_{29}H_{47}N_4O_5$ $[M+H]^+$ 531.3541, found 531.3540

-{[1-(1-Oxyl-2,2,6,6-tetramethylpiperidin-4-yl)][(1-oxyl-2,2,6,6- tetramethylpiperidin-4-yl)carbamoyl]amino] methyl}benzoic acid (**bTureaB**)

A solution of ester 5 (1 equiv) and NaOH (5 eq) in a 1:1 mixture EtOH:H_2O was stirred at 80 °C for 4 h. It was then allowed to cool to room temperature. At 0 °C, a 6 M HCl solution was added until the pH was around 2-3 and the product precipitated. It was then filtered off, washed with EtOH and H_2O and dried to give the desired acid (51 %).

HRMS-ESI: m/z calcd for $C_{27}H_{43}N_4O_5$ $[M+H]^+$ 503.3228, found 503.3226

PyPolB

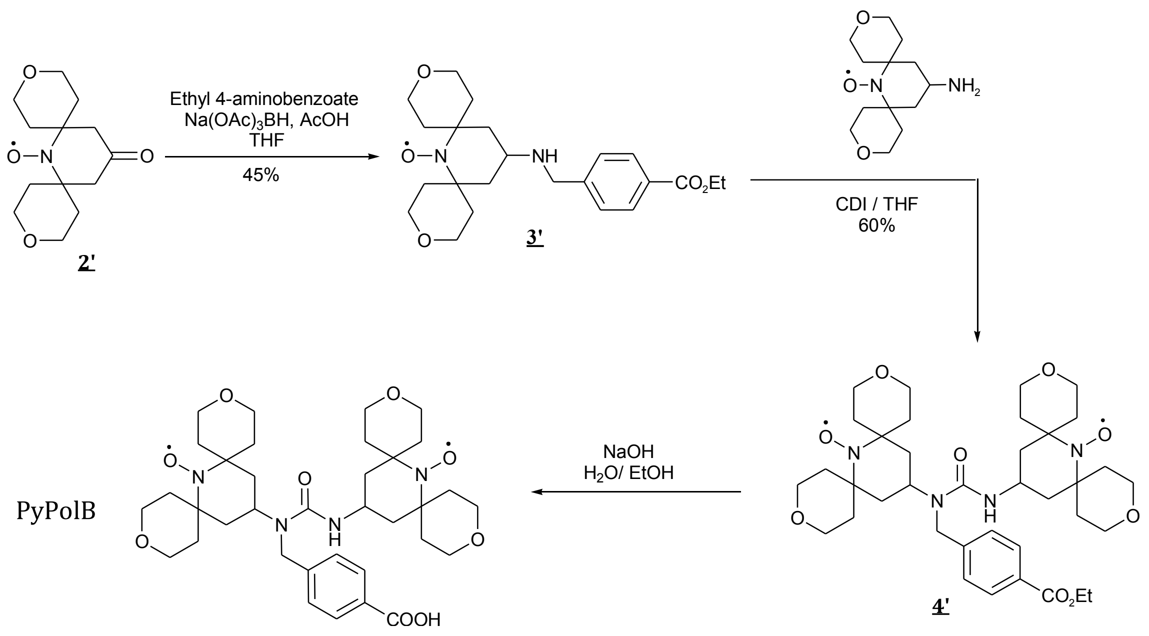

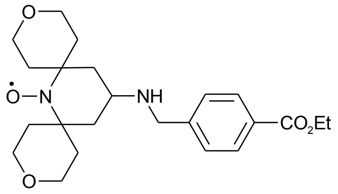

To a solution of ketone **2**' (100mg, 0.39 mmol) and ethyl 4-aminobenzoate (70mg, 0.39 mmol) in dry THF (4 mL) were added Na_2SO_4 and AcOH to adjust pH at 6-7 and the solution was stirred at room temperature for 2 h. Then, $Na(OAc)_3BH$ (150mg, 0.71 mmol) was added portionwise and the reaction was stirred at 25°C for 6 h. The mixture was concentrated under reduced pressure. The residue was solubilized in CH_2Cl_2, washed with a saturated aqueous solution of K_2CO_3 35% (10mL), dried on Na_2SO_4, concentrated under reduced pressure and the crude product was purified by SiO_2 column chromatography with CH_2Cl_2/EtOH (9/1) as eluent to give the desired compound **3'** (73 mg, 0.18 mmol, 45%). ESI-MS: m/z = 418 [M+H] +; 440 [M+Na]+

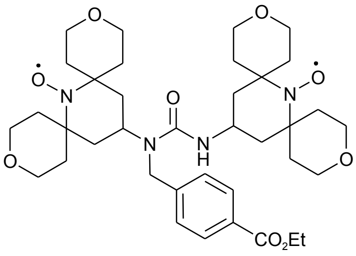

Under argon, to a solution of carbonyldiimidazole (CDI) (107 mg, 0.66 mmol) in THF (2 mL) was added a solution of 4-amino piperidine derivative (145 mg, 0.57 mmol) in THF (2 mL) at 0 °C. The solution was stirred 30 min at room temperature. Then a solution of amine **3’** (230 mg, 0.57 mmol) was added and the resulting mixture was stirred at 70 °C for 6 h. The solution was concentrated under reduced pressure and the residue was solubilized in CH_2Cl_2 and washed with a saturated aqueous solution of $NaHCO_3$ (30 mL). The organic phase was dried on Na_2SO_4, concentrated under reduced pressure and the residue was purified by SiO_2 column chromatography with CH_2Cl_2/EtOH (90/10) as eluent to afford **4**’ as a red solid (240 mg, 0.34 mmol, 60 %). ESI-MS: m/z = 699 [M+H] +; 721 [M+Na]+

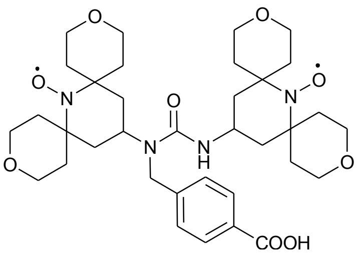

PyPolB

To a solution of **4’** (225 mg, 0.33 mmol) in a mixture of water/ethanol 1:1 (3 mL) was added NaOH in pellets (66 mg, 1.65 mmol). The solution was stirred at 40°C during 1h, then 1N HCl aqueous solution (5 mL) was added to obtain pH = 7. The mixture was concentrated under reduced pressure and the residue was purified by SiO_2 column chromatography with CH_2Cl_2/EtOH (80/20) to afford **PyPolB** as a red solid (200 mg, 0.30 mmol, 95%). HRMS-ESI: m/z calcd for $C_{35}H_{50}N_4O_9^{\bullet}$ [M+H]+671.3651, found 670.3578

Synthesis of the dendrimers

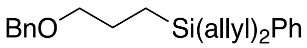

D1[G1](allyl)

Under N_2, to a solution of allylbenzyl ether (2.96 g, 20.0 mmol) and dichlorophenylsilane (3.90 g, 22.0 mmol) in dry toluene (20.0 mL) was added platinum(0)-1, 3-divinyl-1, 1, 3, 3-tetra-methyldisiloxane complex solution (200 μL, Pt ~ 2% in xylene). The mixture was stirred for 16 h at 60 °C, then toluene and excess dichlorophenylsilane were removed *in vacuo*. Dry THF (40.0 mL) was then added to the residue under N_2, and allylmagnesium chloride solution (22.0 mL, 44.0 mmol, 2 M in THF) was added into the mixture at 0 °C via syringe pump at a rate of 1 mL/min. The mixture was warmed up to room temperature and further stirred for 16 h at 50 °C. After cooling to room temperature, the mixture was poured into cold saturated ammonium chloride aqueous solution and extracted with pentane. The organic layer was collected, washed with water and brine, and dried over magnesium sulfate. After evaporation of solvent *in vacuo*, the residue was chromatographed over silica gel (cyclohexane/Et_2O = 100/1) and distilled with Kugelrohr *in vacuo* (200 °C, 0.1 mbar) to afford **D1[G1](allyl)** as colorless oil (3.37 g, 50%). ^1H NMR (300 MHz, $CDCl_3$): δ 0.84-0.90 (m, 2H), 1.62-1.73 (m, 2H), 1.86 (d, *J* = 8.0 Hz, 4H), 3.43 (t, *J* = 6.8 Hz, 2H), 4.48 (s, 2H), 4.85-4.94 (m, 4H), 5.72-5.87 (m, 2H), 7.27-7.37 (m, 8H), 7.49-7.52 (m, 2H). ^{13}C NMR (75MHz, $CDCl_3$): δ 7.92, 19.89, 23.74, 72.78, 73.05, 114.06, 127.47, 127.61, 127.77, 128.33, 129.22, 134.08, 134.16, 135.73, 138.60. HRMS (MALDI) *m/z* = 359.1800 [$C_{22}H_{28}OSi + Na^+$].

D1[G1](allyl)_¹H NMR spectrum

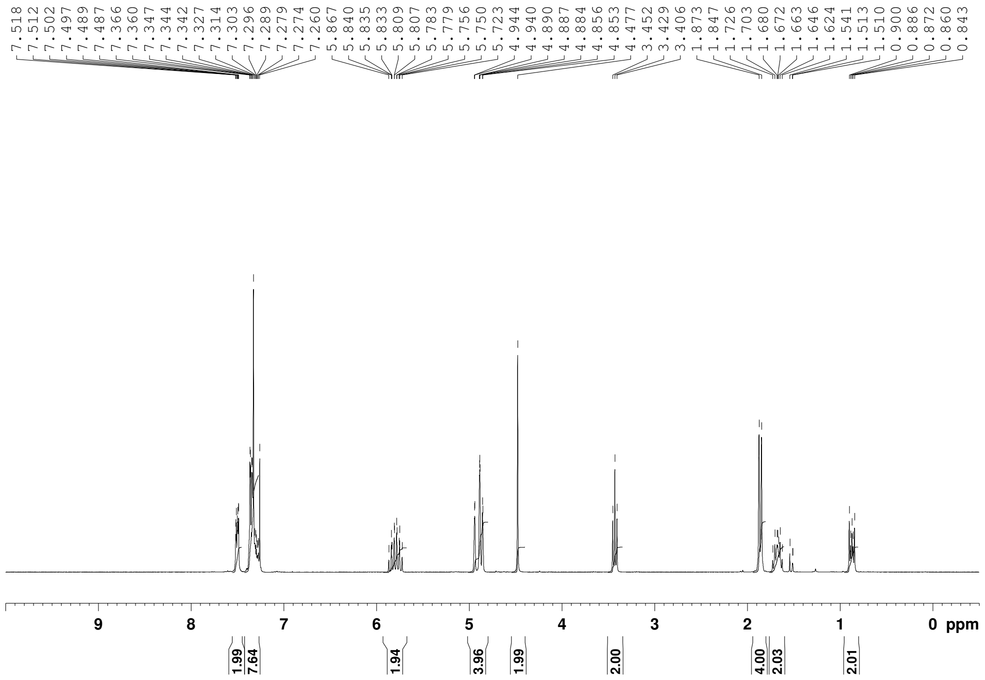

D1[G1](allyl)_¹³C NMR spectrum

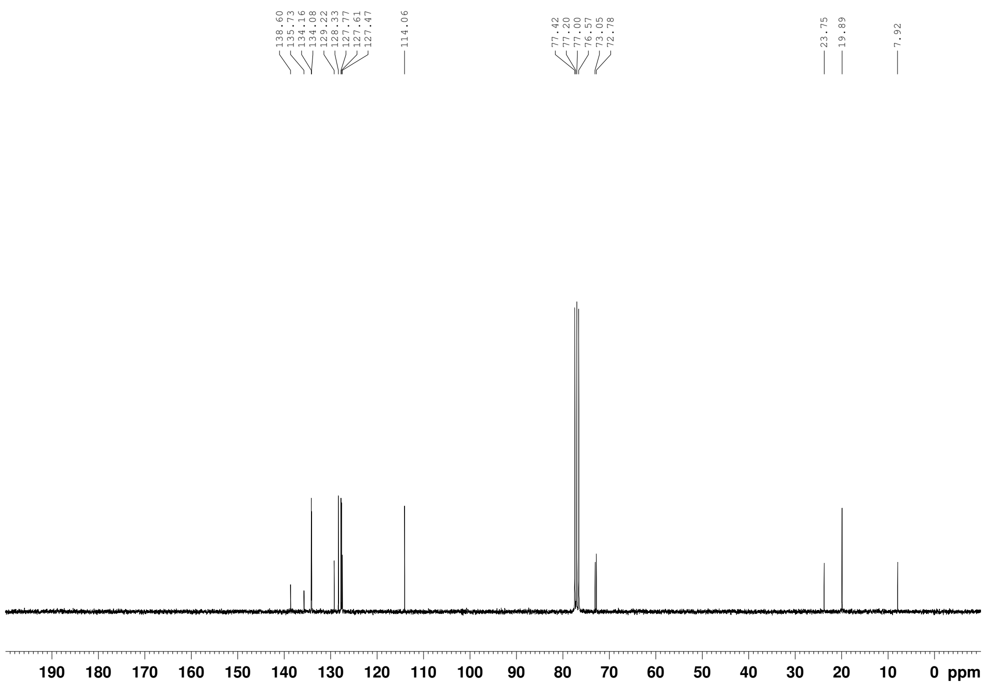

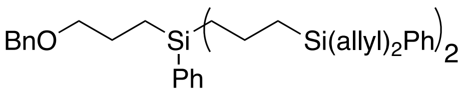

D1[G2](allyl)

Under N_2, to a solution of **D1[G1](allyl)** (2.02 g, 6.00 mmol) and dichlorophenylsilane (2.34 g, 13.2 mmol) in dry toluene (15.0 mL) was added platinum(0)-1, 3-divinyl-1, 1, 3, 3-tetra-methyldisiloxane complex solution (120 μL, Pt ~ 2% in xylene). The mixture was stirred for 16 h at 60 °C, then toluene and excess dichlorophenylsilane were removed *in vacuo*. Dry THF (30.0 mL) was then added to the residue under N_2, and allylmagnesium chloride solution (13.2 mL, 26.4 mmol, 2 M in THF) was added into the mixture at 0 °C via syringe pump at a rate of 1 mL/min. The mixture was warmed up to room temperature and further stirred for 16 h at 50 °C. After cooling to room temperature, the mixture was poured into cold saturated ammonium chloride aqueous solution and extracted with pentane. The organic layer was collected, washed with water and brine, and dried over magnesium sulfate. After evaporation of solvent *in vacuo*, the residue was chromatographed over silica gel (cyclohexane/Et_2O = 50/1) to afford **D1[G2](allyl)** as colorless oil (3.40 g, 79%). ^1H NMR (300 MHz, $CDCl_3$): δ 0.69-0.75 (m, 2H), 0.80-0.91 (m, 8H), 1.34-1.45 (m, 4H), 1.50-1.60 (m, 2H), 1.78 (d, *J* = 8.0 Hz, 8H), 3.36 (t, *J* = 6.9 Hz, 2H), 4.45 (s, 2H), 4.81-4.87 (m, 8H), 5.66-5.80 (m, 4H), 7.29-7.39 (m, 16H), 7.42-7.45 (m, 4H). ^{13}C NMR (75MHz, $CDCl_3$): δ 8.54, 16.58, 17.13, 18.05, 20.08, 24.06, 72.77, 73.22, 113.87, 127.44, 127.60, 127.68, 127.71, 128.32, 128.78, 129.10, 134.01, 134.10, 134.22, 136.06, 137.25, 138.65. HRMS (MALDI) *m/z* = 735.3844 [$C_{46}H_{60}OSi_3 + Na^+$].

D1[G2](allyl)_¹H NMR spectrum

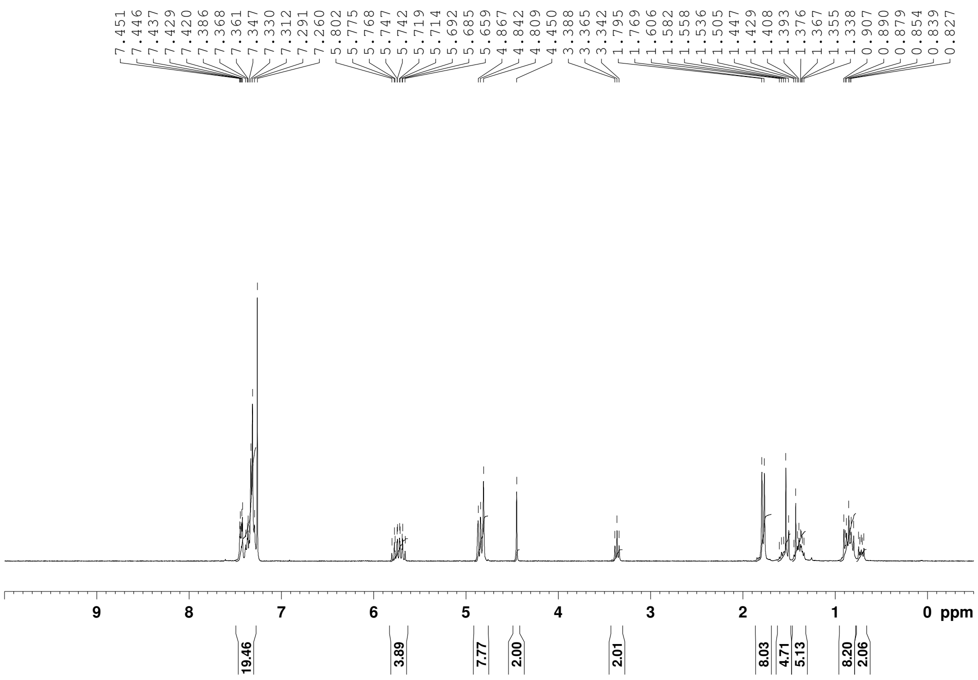

D1[G2](allyl)_¹³C NMR spectrum

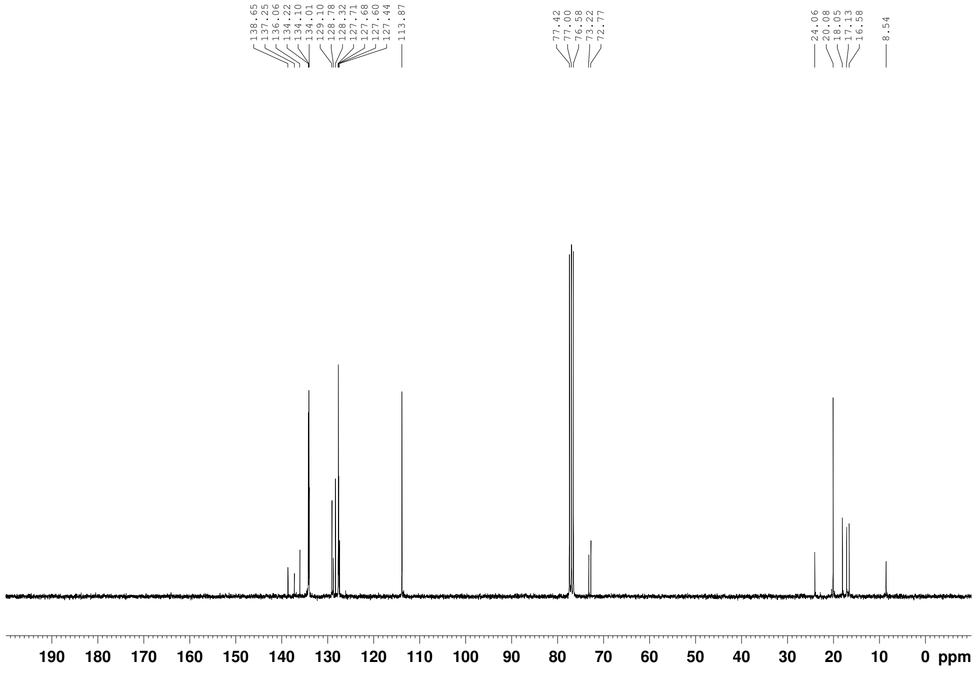

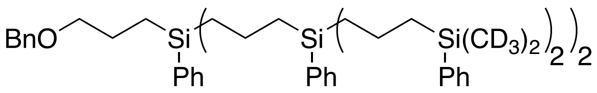

D1[G3]OBn

Under N_2, to a solution of **D1[G2](allyl)** (1.50 g, 2.10 mmol) and dichlorophenylsilane (1.64 g, 9.25 mmol) in dry toluene (15.0 mL) was added platinum(0)-1, 3-divinyl-1, 1, 3, 3-tetra-methyldisiloxane complex solution (20.0 μL, Pt ~ 2% in xylene). The mixture was stirred for 16 h at 60 °C, then toluene and excess dichlorophenylsilane were removed *in vacuo*. Dry THF (30.0 mL) was then added to the residue under N_2, and methyl-d3-magnesium iodide solution (18.5 mL, 18.5 mmol, 1 M in Et_2O) was added into the mixture at 0 °C via syringe pump at a rate of 1 mL/min. The mixture was warmed up to room temperature and further stirred for 16 h at 50 °C. After cooling to room temperature, the mixture was poured into cold saturated ammonium chloride aqueous solution and extracted with pentane. The organic layer was collected, washed with water and brine, and dried over magnesium sulfate. After evaporation of solvent *in vacuo*, the residue was chromatographed over silica gel (cyclohexane/Et_2O = 40/1) to afford **D1[G3]OBn** as colorless sticky oil (2.10 g, 78%). ^1H NMR (300 MHz, $CDCl_3$): δ 0.65-0.85 (m, 26H), 1.23-1.38 (m, 12H), 1.48-1.59 (m, 2H), 3.34 (t, *J* = 6.9 Hz, 2H), 4.43 (s, 2H), 7.25-7.32 (m, 32H), 7.42-7.45 (m, 8H). ^{13}C NMR (75MHz, $CDCl_3$): δ -3.82 (m, CD_3), 8.46, 17.10, 17.24, 17.45, 18.34, 18.41, 20.27, 24.05, 72.74, 73.29, 127.43, 127.58, 127.67, 128.31, 128.56, 128.68, 133.49, 134.00, 134.38, 137.45, 137.84, 138.67, 139.66. HRMS (MALDI) *m/z* = 1303.8178 [$C_{78}H_{84}D_{24}OSi_7$ + Na^+].

D1[G3]OBn_^{1}H NMR spectrum

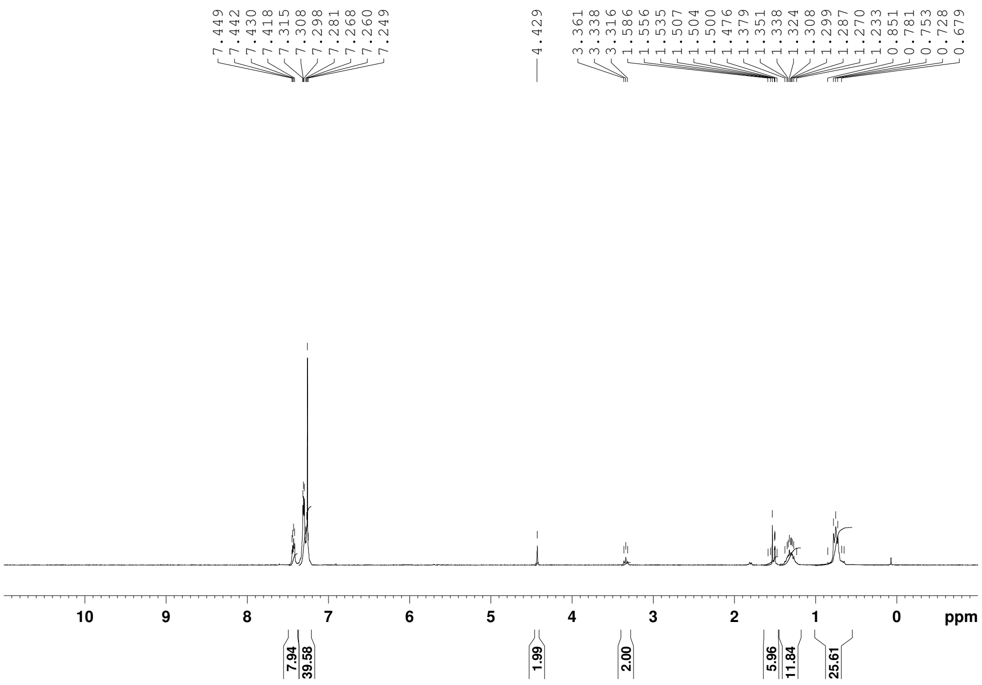

D1[G3]OBn_^{13}C NMR spectrum

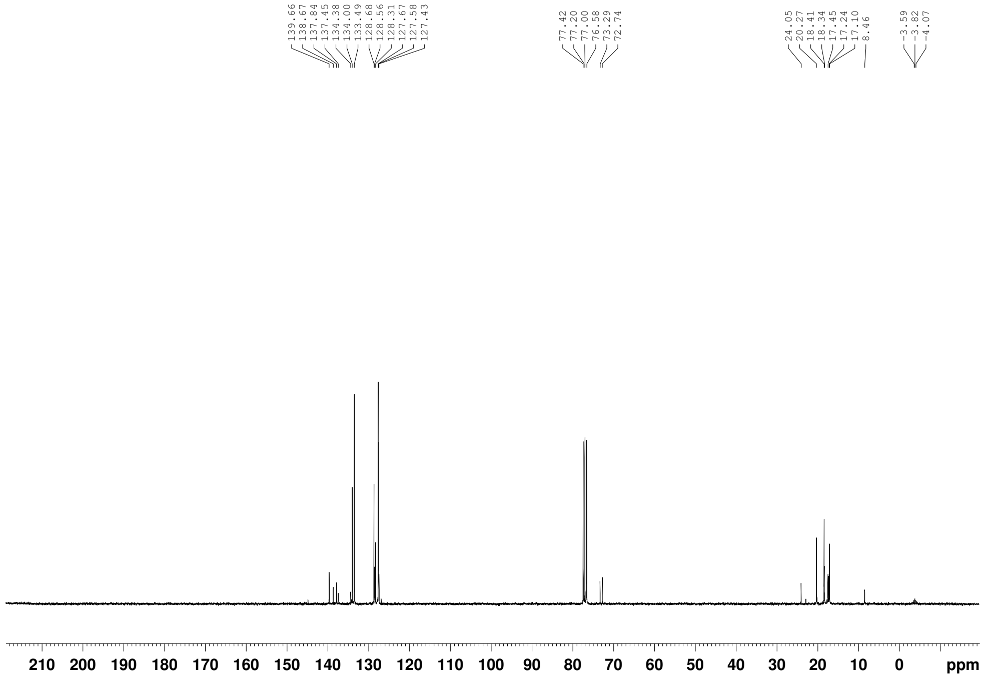

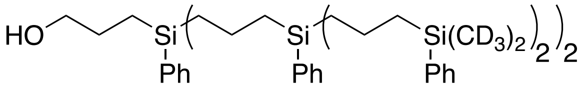

D1[G3]

To a solution of **D1[G3]OBn** (340 mg, 0.270 mmol) in dichloromethane (4.00 mL) was added 2, 3-dichloro-5, 6-dicyano-1, 4-benzoquinone (90.0 mg, 0.400 mmol). The mixture was refluxed for 16 h, then it was cooled to room temperature and poured into cold saturated sodium bicarbonate aqueous solution. The mixture was extracted with dichloromethane, and the organic layer was collected, washed with water and brine, and dried over magnesium sulfate. After evaporation of the solvent *in vacuo*, the residue was chromatographed over silica gel (cyclohexane/EA = 15/1) to afford **D1[G3]** as colorless sticky oil (210 mg, 66%). ^{1}H NMR (300 MHz, $CDCl_3$): δ 0.61-0.67 (m, 2H), 0.73-0.78 (m, 24H), 1.05 (t, J = 5.7 Hz, 1H), 1.23-1.39 (m, 14H), 3.42-3.49 (m, 2H), 7.28-7.34 (m, 27H), 7.42-7.45 (m, 8H). ^{13}C NMR (75MHz, $CDCl_3$): δ -3.59 (m, CD_3), 7.99, 17.10, 17.21, 17.42, 18.33, 18.40, 20.28, 27.10, 65.66, 127.58, 127.67, 128.57, 128.69, 128.76, 133.49, 133.96, 134.01, 134.40, 137.34, 137.85, 139.65. HRMS (MALDI) m/z = 1213.7714 [$C_{71}H_{78}D_{24}OSi_7 + Na^+$].

D1[G3]_^{1}H NMR spectrum

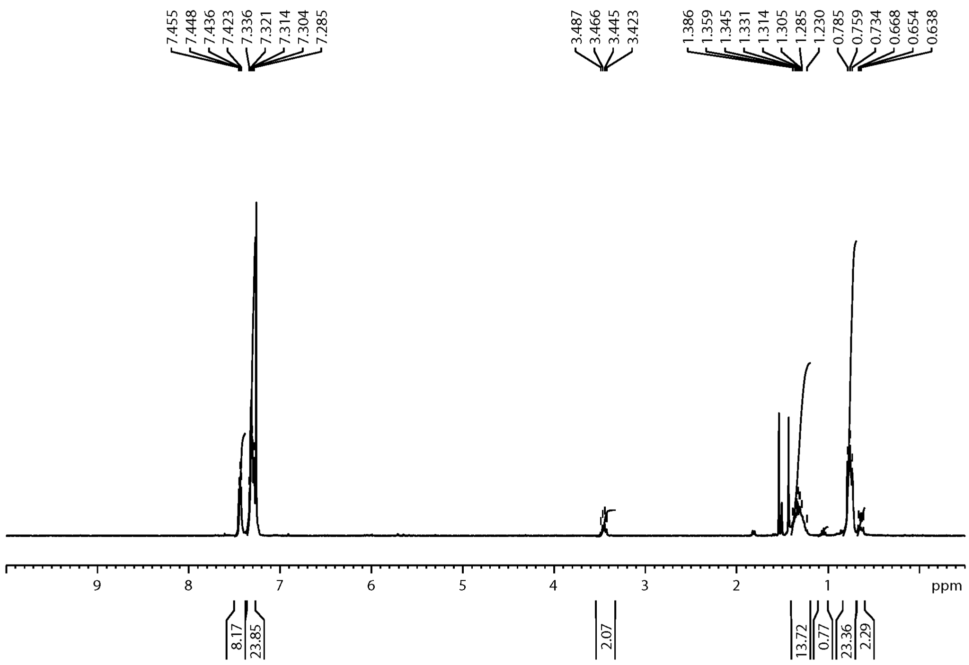

D1[G3]_^{13}C NMR spectrum

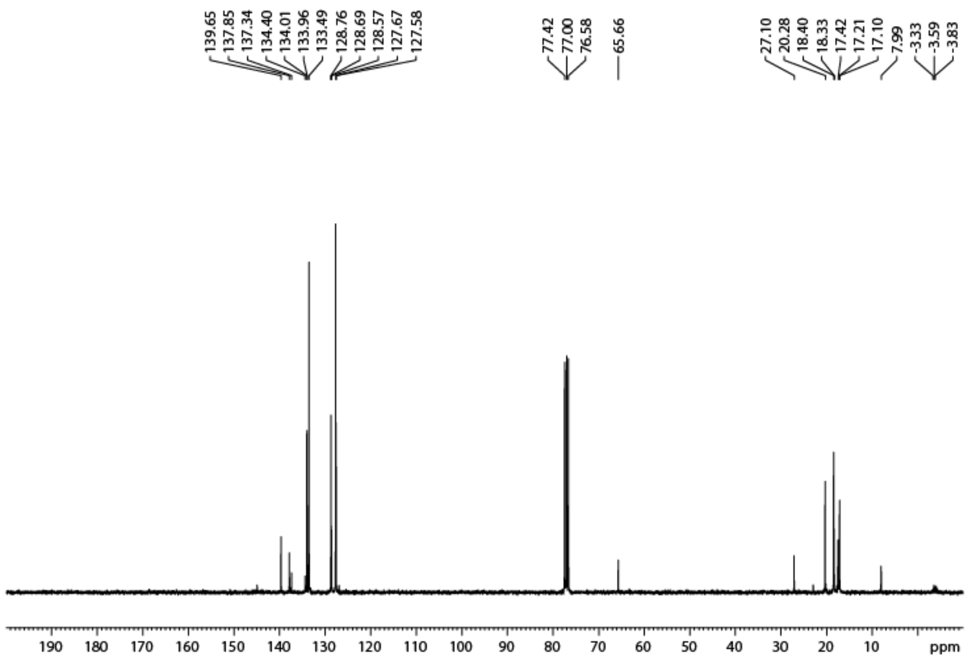

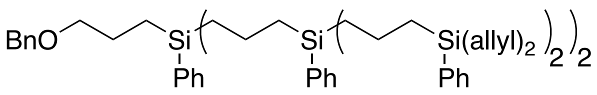

D1[G3](allyl)

Under N_2, to a solution of **D1[G2](allyl)** (2.85 g, 4.00 mmol) and dichlorophenylsilane (3.12 g, 17.6 mmol) in dry toluene (20 mL) was added platinum(0)-1, 3-divinyl-1, 1, 3, 3-tetra-methyldisiloxane complex solution (80 µL, Pt ~ 2% in xylene). The mixture was stirred for 16 h at 60 °C, then toluene and excess dichlorophenylsilane were removed *in vacuo*. Dry THF (40 mL) was then added to the residue under N_2, and allyl magnesium bromide solution (17.6 mL, 35.2 mmol, 2 M in THF) was added into the mixture at 0 °C via syringe pump at a rate of 1 mL/min. The mixture was warmed up to room temperature and further stirred for 16 h at 50 °C. After cooling to room temperature, the mixture was poured into cold saturated ammonium chloride aqueous solution and extracted with pentane. The organic layer was collected, washed with water and brine, and dried over magnesium sulfate. After evaporation of solvent *in vacuo*, the residue was chromatographed over silica gel (cyclohexane/Et_2O = 50/1) to afford **D1[G3](allyl)** as colorless sticky oil (5.10 g, 87%). ^1H NMR (300 MHz, $CDCl_3$): δ 0.67-0.91 (m, 26H), 1.27-1.44 (m, 12H), 1.49-1.61 (m, 2H), 1.78 (d, *J* = 8.0 Hz, 16H), 3.35 (t, *J* = 6.7 Hz, 2H), 4.44 (s, 2H), 4.81-4.87 (m, 16H), 5.66-5.80 (m, 8H), 7.28-7.33 (m, 32H), 7.42-7.44 (m, 8H). ^{13}C NMR (75MHz, $CDCl_3$): δ 8.37, 16.56, 17.23, 17.38, 18.07, 18.30, 20.06, 22.86, 24.05, 72.73, 73.25, 127.42, 127.61, 127.69, 128.30, 128.63, 128.71, 129.08, 133.99, 134.08, 134.21, 134.37, 136.06, 137.35, 137.56, 138.66. HRMS (MALDI) *m/z* = 1487.7929 [$C_{94}H_{124}OSi_7 + Na^+$].

D1[G3](allyl)_^{1}H NMR spectrum

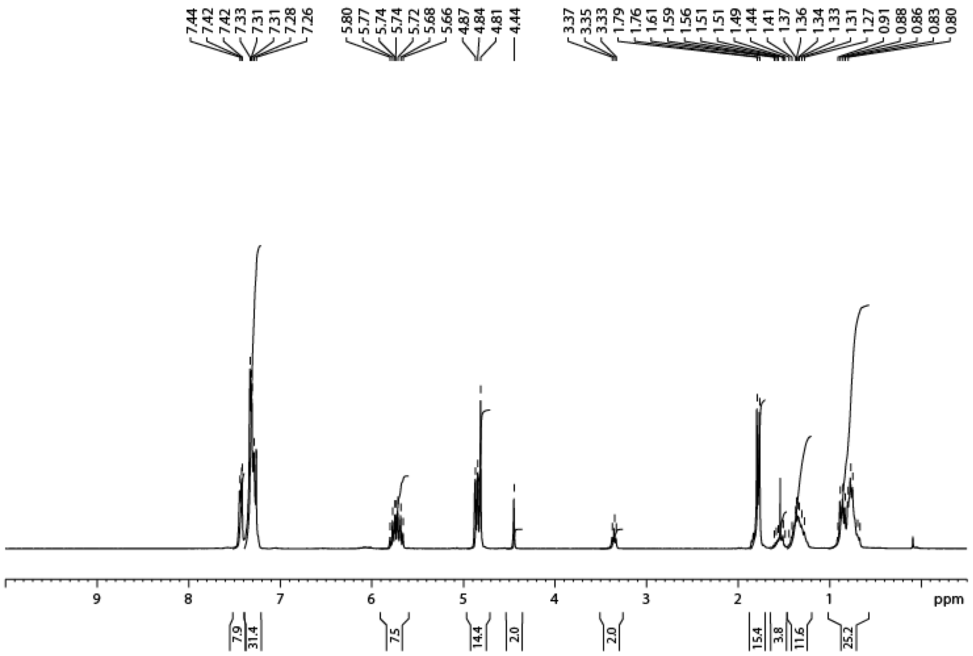

D1[G3](allyl)_^{13}C NMR spectrum

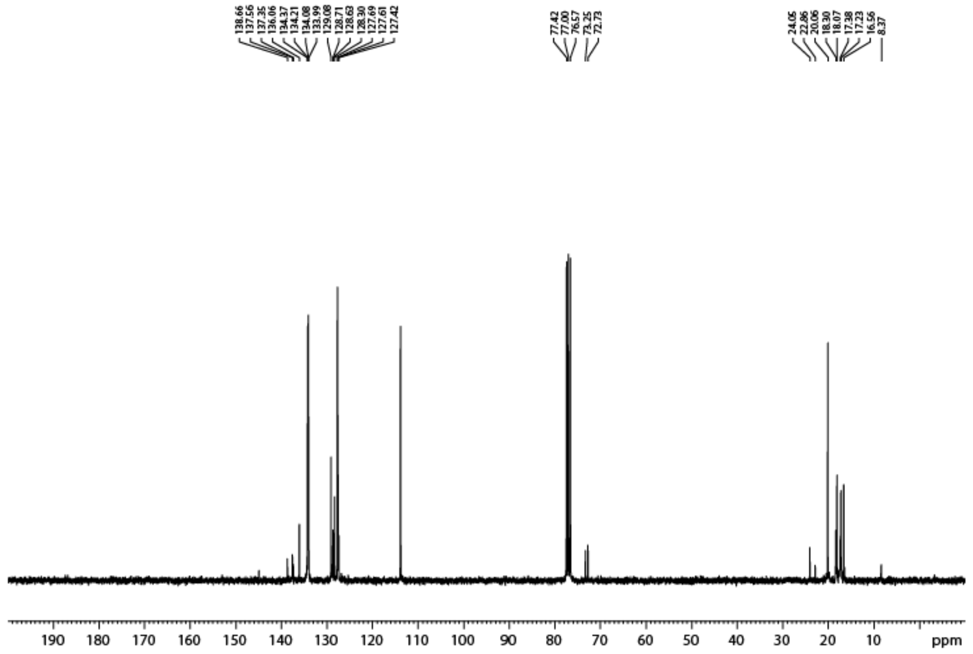

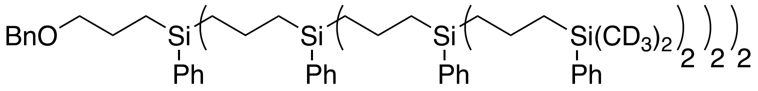

D1[G4]OBn

Under N_2, to a solution of **D1[G3](allyl)** (1.47 g, 1.00 mmol) and dichlorophenylsilane (1.56 g, 8.80 mmol) in dry toluene (15.0 mL) was added a platinum(0)-1, 3-divinyl-1, 1, 3, 3-tetra-methyldisiloxane complex solution (80.0 μL, Pt ~ 2% in xylene). The mixture was stirred for 16 h at 60 °C, then toluene and excess dichlorophenylsilane were removed *in vacuo*. Dry THF (60.0 mL) was then added to the residue under N_2, and methyl-d3-magnesium iodide solution (24.0 mL, 24.0 mmol, 1 M in Et_2O) was added into the mixture at 0 °C via syringe pump at a rate of 1 mL/min. The mixture was warmed up to room temperature and further stirred for 16 h at 50 °C. After cooling to room temperature, the mixture was poured into cold saturated ammonium chloride aqueous solution and extracted with pentane. The organic layer was collected, washed with water and brine, and dried over magnesium sulfate. After evaporation of solvent *in vacuo*, the residue was chromatographed over silica gel (cyclohexane/Et_2O = 40/1) to afford **D1[G4]OBn** as colorless sticky oil (1.99 g, 77%). ^1H NMR (300 MHz, $CDCl_3$): δ 0.71-0.77 (m, 56H), 1.24-1.37 (m, 32H), 3.31 (br, 2H), 4.40 (s, 2H), 7.20-7.30 (m, 64H), 7.42-7.43 (m, 16H). ^{13}C NMR (75MHz, $CDCl_3$): δ -3.81 (m, CD_3), 8.27, 17.07, 17.26, 17.39, 17.49, 17.75, 18.25, 18.34, 18.40, 20.12, 20.27, 22.88, 24.03, 72.72, 73.28, 126.95, 127.41, 127.57, 127.66, 128.30, 128.55, 128.68, 133.49, 133.99, 134.38, 137.41, 137.48, 137.75, 137.83, 138.68, 139.64, 144.78. HRMS (MALDI) *m/z* = 2708.5761 [$C_{158}H_{172}D_{48}OSi_{15}$ + Ag^+].

D1[G4]OBn_^{1}H NMR spectrum

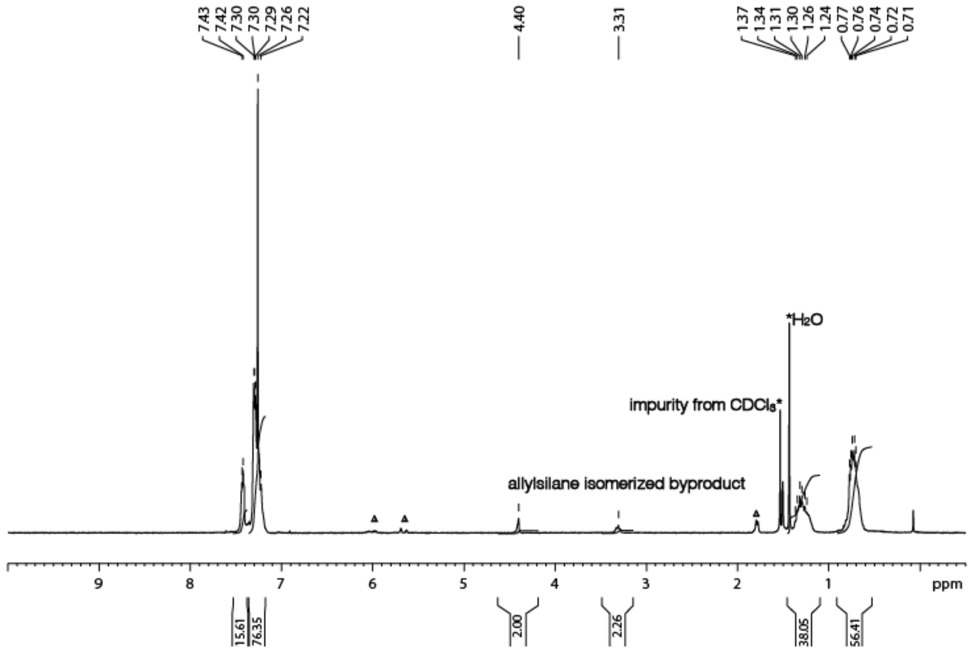

D1[G4]OBn_^{13}C NMR spectrum

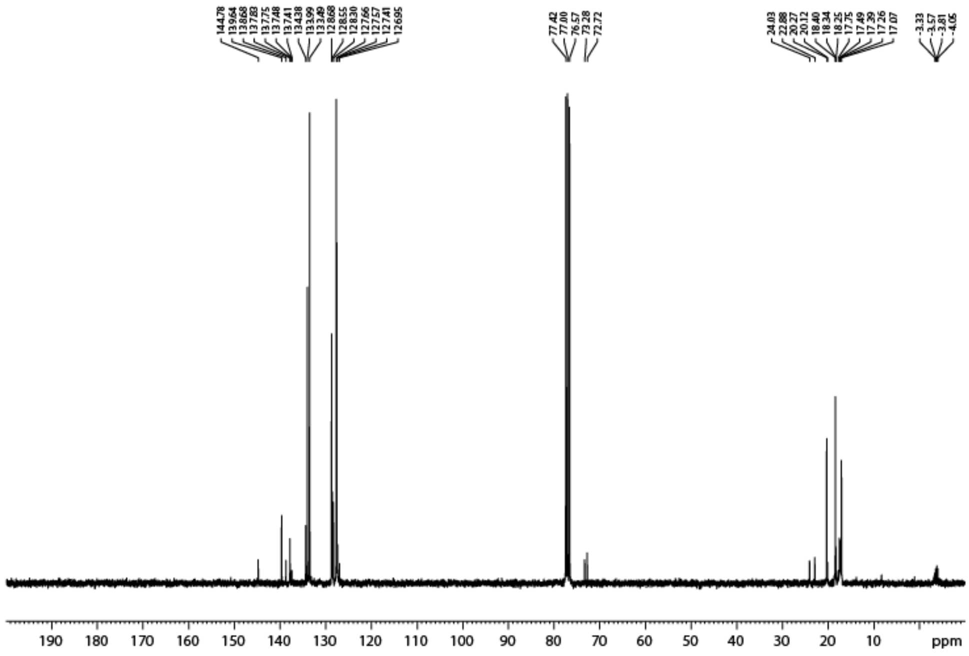

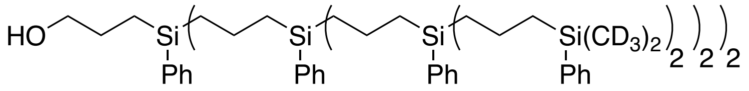

D1[G4]

To a solution of **D1[G4]OBn** (502 mg, 0.19 mmol) in dichloromethane (2 mL) was added 2, 3-dichloro-5, 6-dicyano-1, 4-benzoquinone (44 mg, 0.19 mmol). The mixture was refluxed for 16 h, then it was cooled to room temperature and poured into cold saturated sodium bicarbonate aqueous solution. The mixture was extracted with dichloromethane, and the organic layer was collected, washed with water and brine, and dried over magnesium sulfate. After evaporation of the solvent *in vacuo*, the residue was chromatographed over silica gel (pentane/Et_2O = 10/1, then pentane/EA = 10/1) to afford **D1[G4]** as light yellow sticky oil (280 mg, 59%). ^{1}H NMR (300 MHz, $CDCl_3$): δ 0.71-0.77 (m, 56H), 1.23-1.37 (m, 32H), 3.37 (br, 2H), 5.30 (s, 2H), 7.23-7.31 (m, 60H), 7.41-7.44 (m, 15H). ^{13}C NMR (100MHz, $CDCl_3$): δ -3.82 (m, CD_3), 7.75, 17.06, 17.27, 17.44, 17.48, 17.73, 18.23, 18.33, 18.39, 20.12, 20.27, 22.89, 27.07, 65.60, 126.92, 127.55, 127.57, 127.64, 127.66, 128.55, 128.66, 128.68, 133.48, 133.51, 133.95, 133.98, 134.00, 134.02, 134.38, 137.76, 137.83, 139.64, 144.81. HRMS (MALDI) m/z = 2534.6139 [$C_{151}H_{166}D_{48}OSi_{15}$ + Na^+].

D1[G4]_¹H NMR spectrum

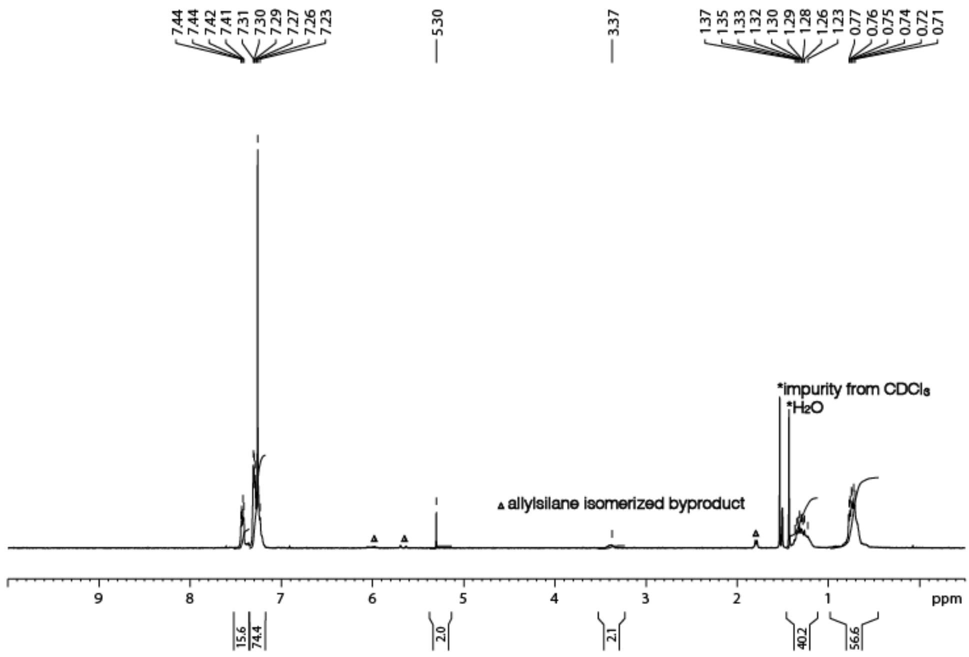

D1[G4]_¹³C NMR spectrum

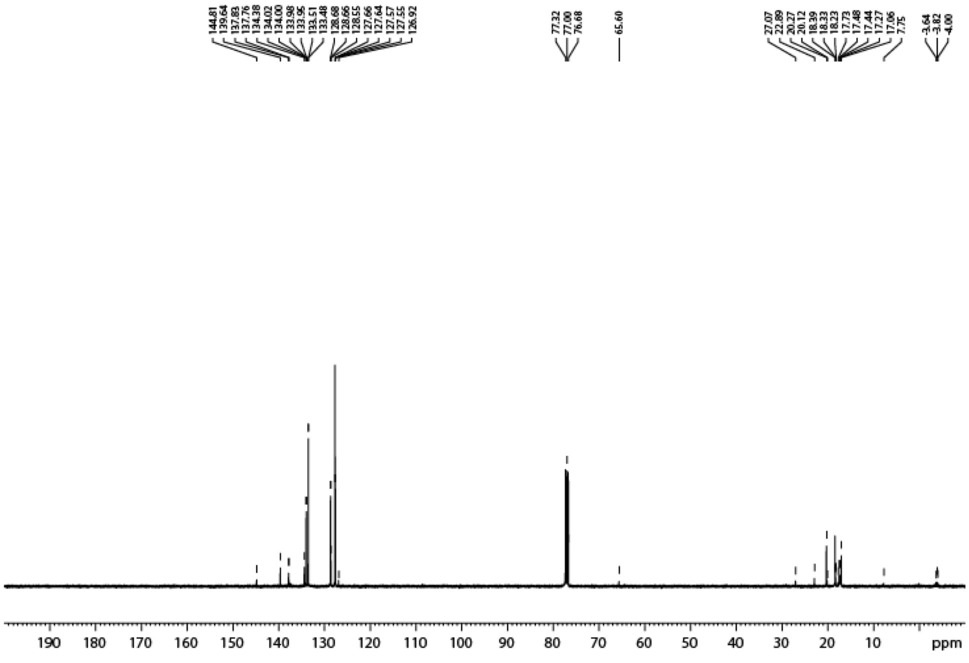

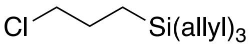

D2[G1](allyl)

Under N_2, allylmagnesium chloride solution (33.0 mL, 66.0 mmol, 2 M in THF) was slowly added via a syringe pump to a solution of trichloro(3-chloropropyl)silane (4.23 g, 20.0 mmol) in dry diethyl ether (60.0 mL) within 90 min at 0 °C. The mixture was warmed to room temperature. After stirring for 4 hours, it was poured into cold saturated ammonium chloride aqueous solution. The mixture was twice extracted with diethyl ether, the organic layers were collected, washed with water and brine and dried over magnesium sulfate. After evaporation of solvent *in vacuo*, the residue was flash chromatographed over silica gel (pure pentane) to afford **D2[G1](allyl)** as colorless oil (4.46 g, quantitative yield). ^{1}H NMR (300 MHz, $CDCl_3$): δ 0.68-0.73 (m, 2H), 1.61 (dt, *J* = 8.1 and 1.1 Hz, 6H), 1.75-1.85 (m, 2H), 3.49 (t, *J* = 6.9 Hz, 2H), 4.87-4.94 (m, 6H), 5.71-5.85 (m, 3H) ^{13}C NMR (75MHz, $CDCl_3$): δ 9.24, 19.47, 27.19, 47.78, 113.95, 133.93. HRMS (MALDI) m/z = 335.0146 [$C_{12}H_{21}ClSi + Ag^+$].

D2[G1](allyl)_¹H NMR spectrum

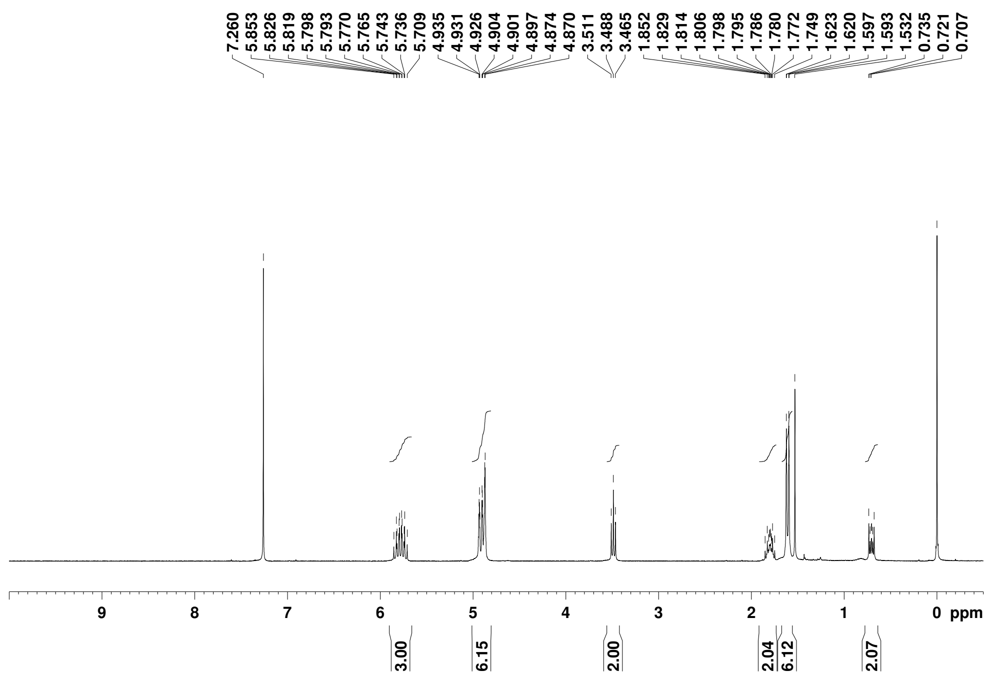

D2[G1](allyl)_¹³C NMR spectrum

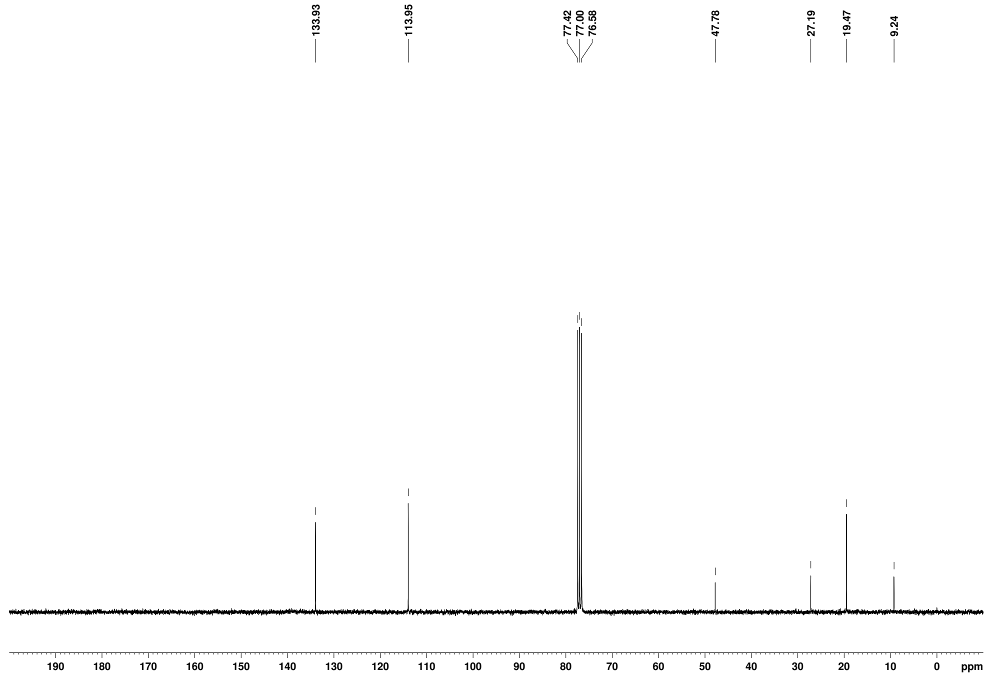

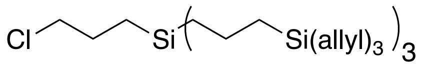

D2[G2](allyl)

Under N_2, bis(tetrabutylammonium) hexachloroplatinate (44.0 mg, 0.050 mmol) was added to a solution of **D2[G1](allyl)** (2.29 g, 10.0 mmol) in a mixture of dry diethyl ether (10 mL) and dry dichloromethane (20 mL). The mixture was stirred for 30 min, then trichlorosilane (4.47 g, 33.0 mmol) was added. The flask was equipped with a calcium chloride tube, and the nitrogen inlet was closed. The mixture was stirred vigorously until ^{1}H NMR suggested full conversion of all allyl end groups, usually after 1 to 2 days reaction time. Solvents and excess trichlorosilane were removed *in vacuo*, and dry diethyl ether (40mL) was introduced to dissolve the residue. To this mixture was slowly added allylmagnesium chloride solution (49.5 mL, 99.0 mmol, 2 M in THF) via a syringe pump within 120 min at 0 °C. The mixture was warmed to room temperature. After stirring for 4 hours, it was poured into cold saturated ammonium chloride aqueous solution. The mixture was twice extracted with diethyl ether, and the organic layers were collected, washed with water and brine and dried over magnesium sulfate. After evaporation of solvent *in vacuo*, the residue was flash chromatographed over silica gel (pentane to pentane/diethyl ether = 50/1 to pentane/diethyl ether = 20/1) to afford **D2[G2](allyl)** as colorless oil (5.87 g, 86%). ^{1}H NMR (300 MHz, $CDCl_3$): δ 0.55-0.69 (m, 14H), 1.29-1.40 (m, 6H), 1.59 (dt, *J* = 8.1 and 1.0 Hz, 6H), 1.68-1.78 (m, 2H), 3.50 (t, *J* = 6.9 Hz, 2H), 4.85-4.92 (m, 18H), 5.71-5.86 (m, 9H) ^{13}C NMR (75MHz, $CDCl_3$): δ 10.16, 16.63, 17.35, 18.18, 19.71, 27.78, 48.06, 113.57, 137.39. HRMS (MALDI) *m/z* = 791.3210 [$C_{39}H_{69}ClSi_4$ + Ag^+].

D2[G2](allyl)_¹H NMR spectrum

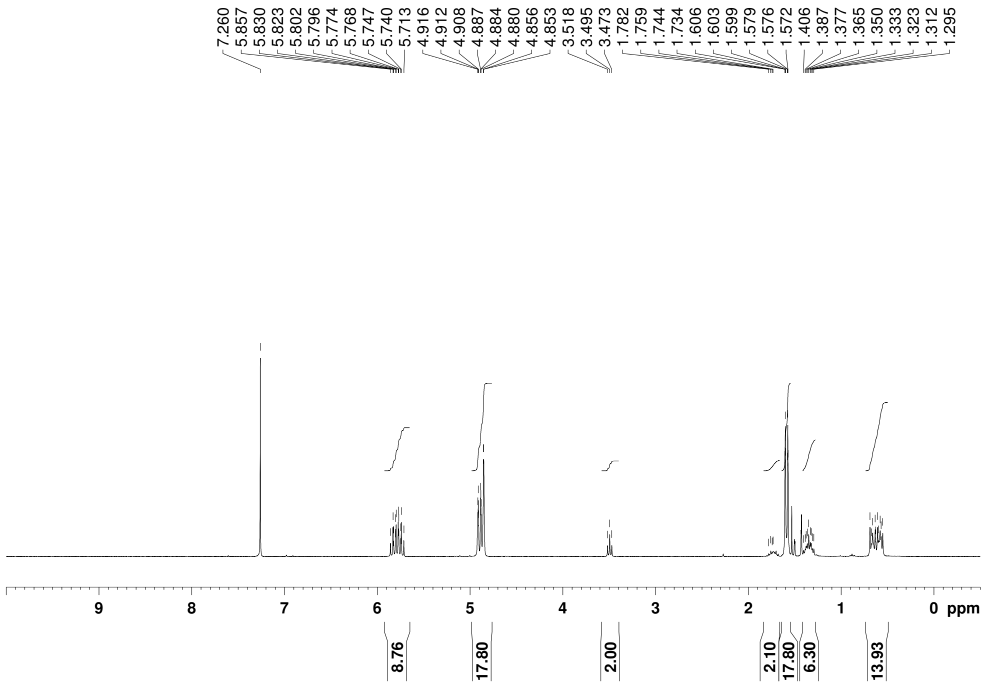

D2[G2](allyl)_¹³C NMR spectrum

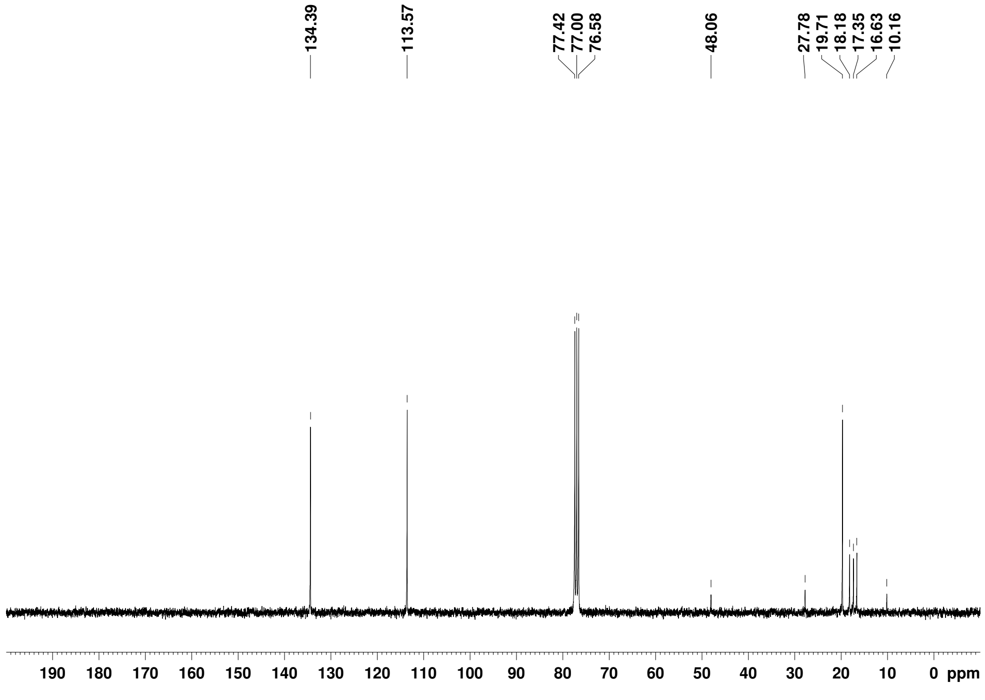

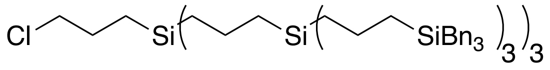

D2[G3]Cl

Under N_2, bis(tetrabutylammonium) hexachloroplatinate (41.0 mg, 0.046 mmol) was added to a solution of **D2[G2](allyl)** (3.14 g, 4.58 mmol) in a mixture of dry diethyl ether (20 mL) and dry dichloromethane (40 mL). The mixture was stirred for 30 min, then trichlorosilane (6.14 g, 45.3 mmol) was added. The flask was equipped with a calcium chloride tube, and the nitrogen inlet was closed. The mixture was stirred vigorously until ^{1}H NMR suggested ful conversion of all allyl end groups, usually after 3 to 4 days reaction time. Solvents and excess trichlorosilane were removed *in vacuo*, and diethyl ether (150mL) was introduced to dissolve the residue. To this mixture was slowly added benzylmagnesium chloride solution (64.0 mL, 128 mmol, 2 M in THF) via a syringe pump within 150 min at 0 °C. The mixture was warmed to room temperature. After stirring for 5 hours, it was poured into cold saturated ammonium chloride aqueous solution. The mixture was three times extracted with diethyl ether, and the organic layers were collected, washed with water and brine and dried over magnesium sulfate. After evaporation of solvent *in vacuo*, the residue was flash chromatographed over silica gel (pentane/diethylether/dichloromethane = 18/1/1 then 8/1/1) to afford **D2[G3]Cl** as light yellow sticky oil (14.5 g, 93%). ^{1}H NMR (500 MHz, $CDCl_3$): δ 0.28-0.31 (m, 18H), 0.38-0.42 (m, 6H), 0.49-0.52 (m, 18H), 0.63-0.66 (m, 8H), 1.02-1.09 (m, 18H), 1.19-1.26 (m, 6H), 1.68-1.74 (m, 2H), 2.09 (s, 54H), 3.28 (t, *J* = 6.6 Hz, 2H), 6.92-6.93 (m, 54H), 7.03-7.06 (m, 27H), 7.15-7.18 (m, 54H) ^{13}C NMR (125MHz, $CDCl_3$): δ 139.29, 128.41, 128.32, 124.29, 48.01, 27.98, 21.89, 18.58, 18.08, 17.97, 17.46, 16.94, 9.97. HRMS (MALDI) *m/z* = 3510.6627 [$C_{228}H_{267}ClSi_{13}$ + Ag^+].

D2[G3]Cl_^{1}H NMR spectrum

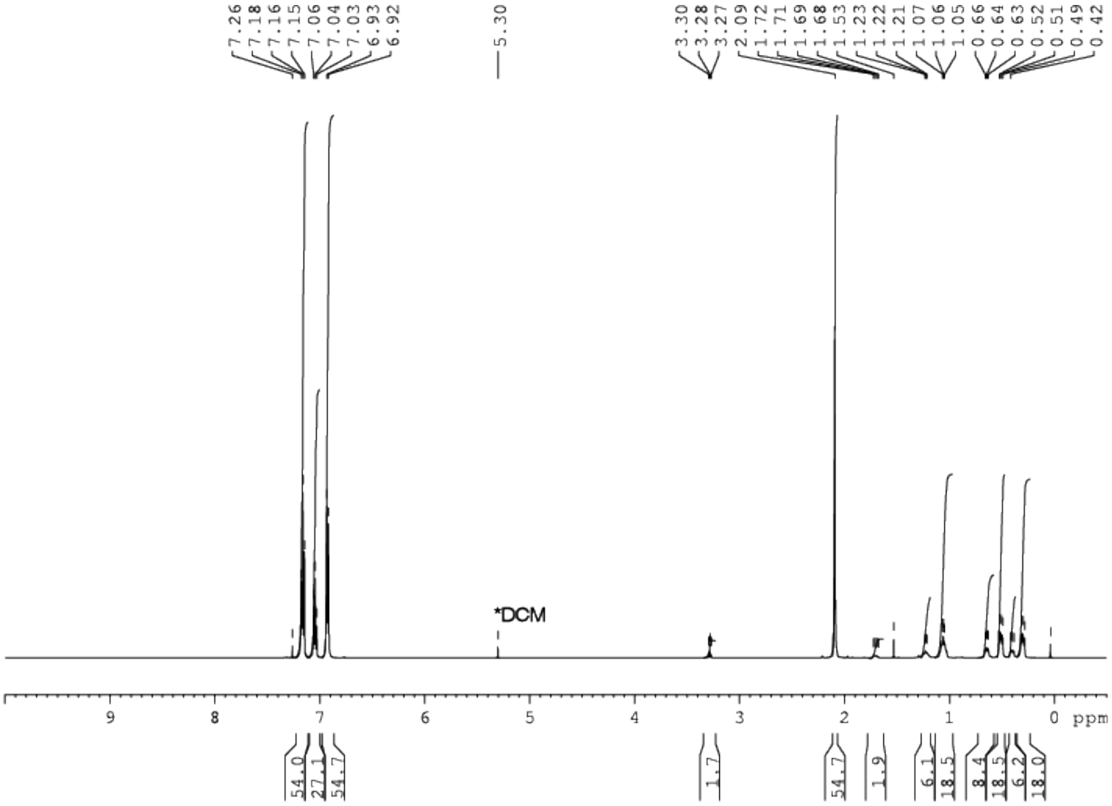

D2[G3]Cl_^{13}C NMR spectrum

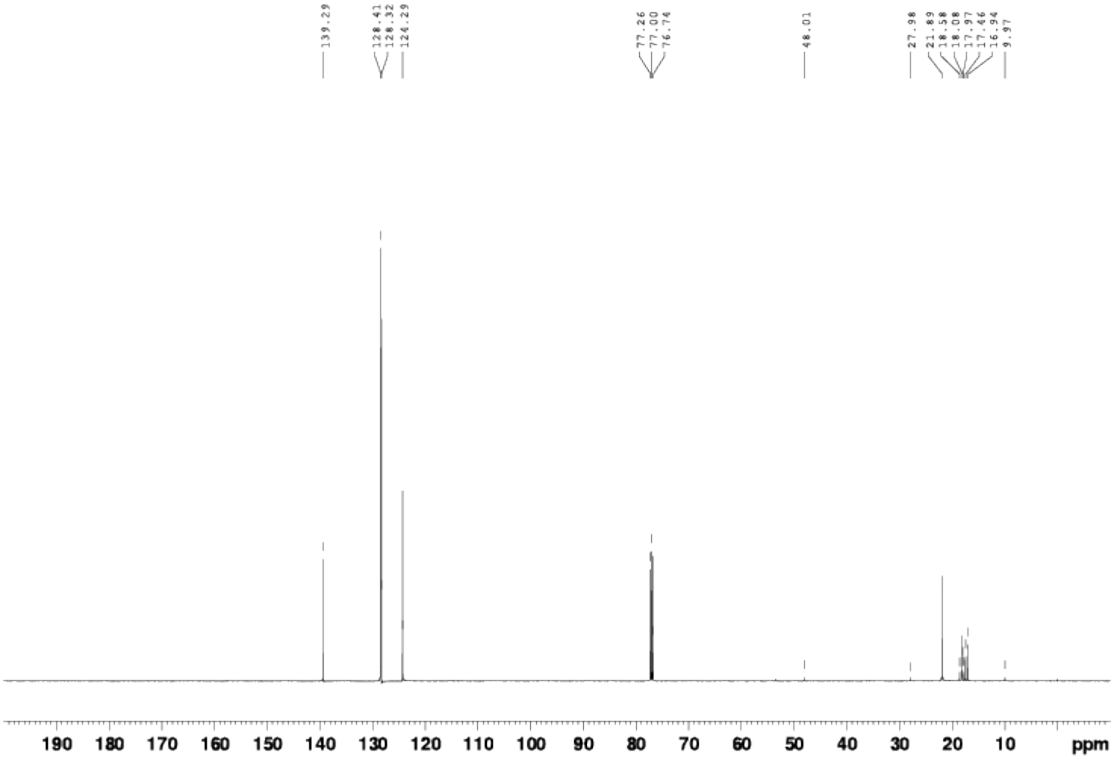

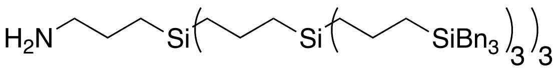

D2[G3]

Under N_2, sodium azide (115 mg, 1.77 mmol) and sodium iodide (8.8 mg, 0.059 mmol) were added to a solution of **D2[G3]Cl** (2.00 g, 0.590 mmol) in dry dimethylformamide (10 mL). The mixture was heated to 100 °C. After stirring for 2 days, the mixture was cooled to room temperature and poured into water. The aqueous layer was three times extracted with diethyl ether, and the organic layers were collected, washed with water and brine, and dried over magnesium sulfate. Next, solvents were removed *in vacuo*. THF (20 mL) was introduced to dissolve the residue, and to this solution was added triphenylphosphine (464 mg, 1.77 mmol). After stirring for 1 day, a few drops of water were added to the mixture, and stirring was continued for 16 hours. The mixture was poured into water, and the aqueous layer was three times extracted with diethyl ether. The organic layers were collected, washed with water and brine, and dried over magnesium sulfate. After evaporation of solvent *in vacuo*, the residue was chromatographed over silica gel (pentane/diethyl ether = 40/1 to 20/1 to 10/1, then pentane/diethyl ether/dichloromethane = 2/1/1) to afford **D2[G3]** as light yellow sticky oil (0.80 g, 40%). ^{1}H NMR (500 MHz, $CDCl_3$): δ 0.26-0.29 (m, 18H), 0.37-0.40 (m, 6H), 0.46-0.50 (m, 20H), 0.61-0.64 (m, 6H), 1.00-1.07 (m, 18H), 1.18-1.24 (m, 6H), 1.31-1.37 (m, 2H), 1.39 (br, 2H), 2.06 (s, 54H), 2.46 (t, J = 6.9 Hz, 2H), 6.89-6.91 (m, 54H), 7.00-7.03 (m, 27H), 7.12-7.15 (m, 54H) ^{13}C NMR (125MHz, $CDCl_3$): δ 139.31, 128.45, 128.34, 124.33, 45.80, 28.50, 21.92, 18.67, 18.21, 18.12, 17.51, 16.97, 9.78. HRMS (MALDI) m/z = 3491.7126 [$C_{228}H_{269}NSi_{13}$ + Ag^+].

D2[G3]_^{1}H NMR spectrum

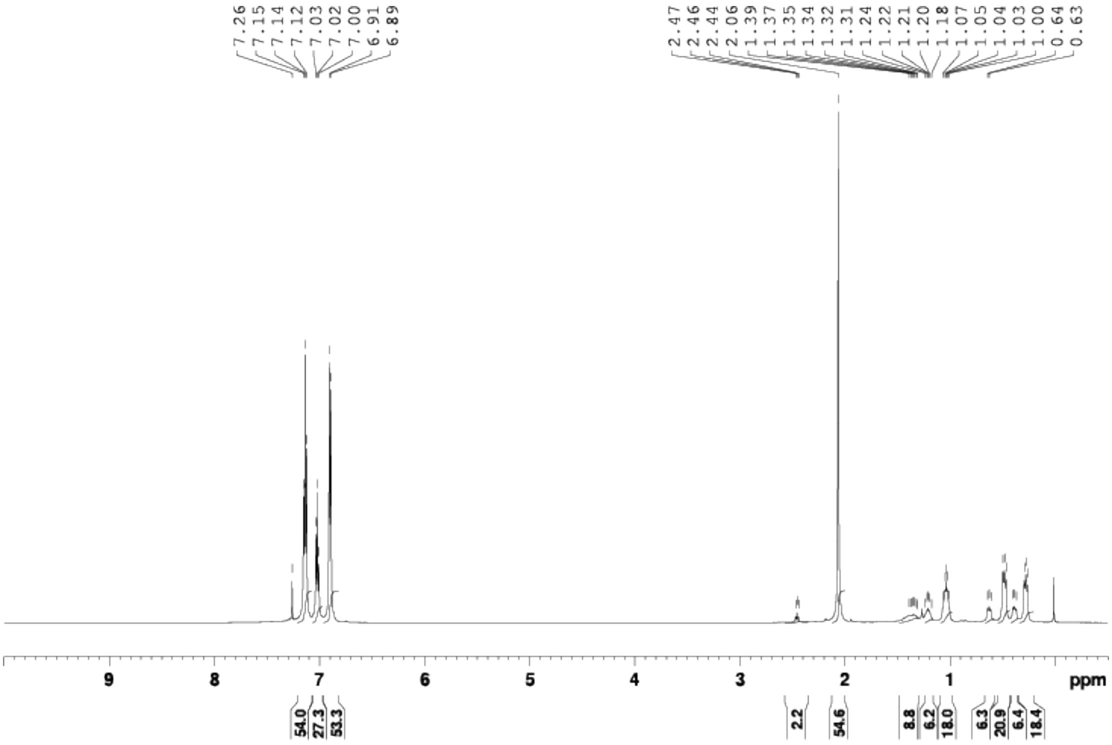

D2[G3]_^{13}C NMR spectrum

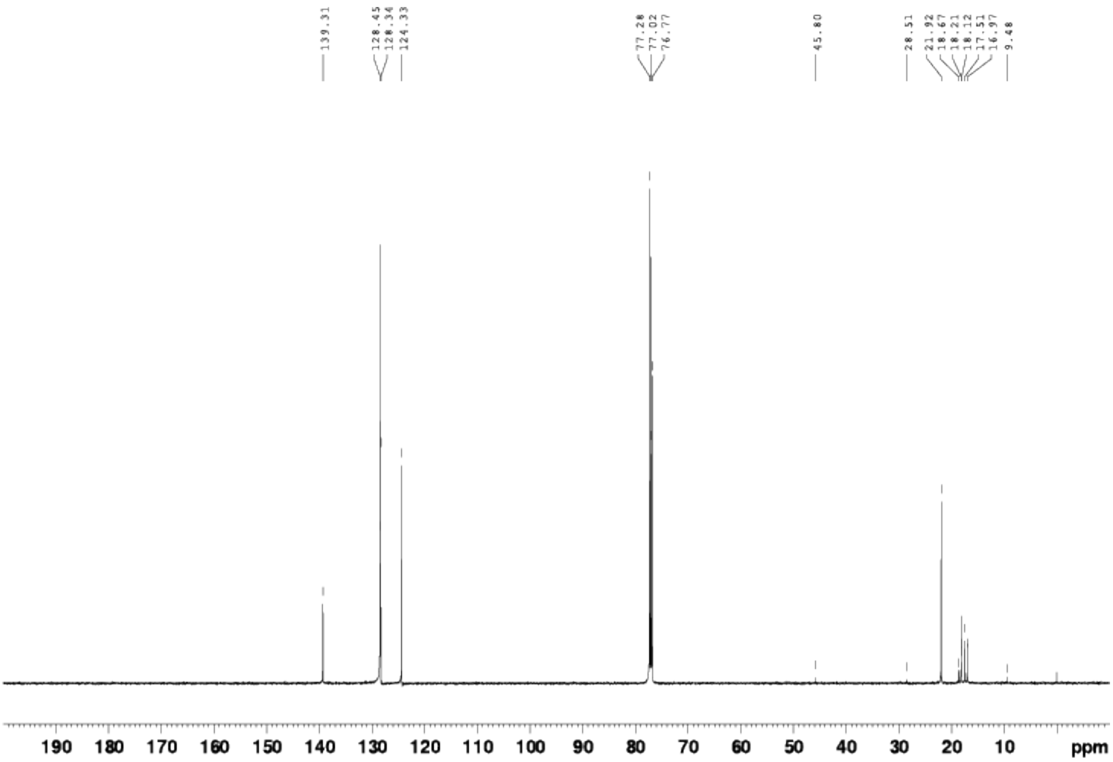

V. References

1. D. Li, G. Kagan, R. Hopson and P. G. Williard, *J. Am. Chem. Soc.*, 2009, **131**, 5627-5634.
2. B. M. Fung, A. K. Khitrin and K. Ermolaev, *J. Magn. Reson.*, 2000, **142**, 97-101.
3. A. J. Rossini, A. Zagdoun, M. Lelli, D. Gajan, F. Rascon, M. Rosay, W. E. Maas, C. Copéret, A. Lesage and L. Emsley, *Chem. Sci.*, 2012, **3**, 108-115.
4. P. Wolf, M. Valla, A. J. Rossini, A. Comas-Vives, F. Nunez-Zarur, B. Malaman, A. Lesage, L. Emsley, C. Copéret and I. Hermans, *Angew. Chem. Int. Ed.*, 2014, **53**, 10179-10183.
5. V. Mougel and C. Coperet, *Chem. Sci.*, 2014, **5**, 2475-2481.
6. M. Pucino, V. Mougel, R. Schowner, A. Fedorov, M. R. Buchmeiser and C. Copéret, *Angew. Chem. Int. Ed.*, 2016, **55**, 4300-4302.
